# An integrated and comparative approach towards identification, characterization and functional annotation of candidate genes for drought tolerance in sorghum (*Sorghum bicolor* (L.) Moench)

**DOI:** 10.1186/s12863-017-0584-5

**Published:** 2017-12-22

**Authors:** Adugna Abdi Woldesemayat, Peter Van Heusden, Bongani K. Ndimba, Alan Christoffels

**Affiliations:** 1 South African Medical Research Council Bioinformatics Unit, South African National Bioinformatics Institute, University of the Western Cape, Private Bag X17, Belleville, 7535 South Africa; 2 Department of Life and Consumer Sciences, College of Agriculture and Environmental Sciences, University of South Africa, UNISA Science Campus, Corner of Christiaan De Wet Road and Pioneer Avenue, Johannesburg, Florida 1710 South Africa; 3 Department of Biotechnology, University of the Western Cape, Private Bag X17, Belleville, Cape Town, 7535 South Africa; 4 Agricultural Research Council, Infruitech-Nietvoorbij, Private Bag X5026, Stellenbosch, 7599 South Africa

**Keywords:** Candidate gene identification, Drought tolerance, Functional genomics, Integrated in silico approach, Genome annotation, *Sorghum bicolor* (L.) Moench

## Abstract

**Background:**

Drought is the most disastrous abiotic stress that severely affects agricultural productivity worldwide. Understanding the biological basis of drought-regulated traits, requires identification and an in-depth characterization of genetic determinants using model organisms and high-throughput technologies. However, studies on drought tolerance have generally been limited to traditional candidate gene approach that targets only a single gene in a pathway that is related to a trait. In this study, we used sorghum, one of the model crops that is well adapted to arid regions, to mine genes and define determinants for drought tolerance using drought expression libraries and RNA-seq data.

**Results:**

We provide an integrated and comparative in silico candidate gene identification, characterization and annotation approach, with an emphasis on genes playing a prominent role in conferring drought tolerance in sorghum. A total of 470 non-redundant functionally annotated drought responsive genes (DRGs) were identified using experimental data from drought responses by employing pairwise sequence similarity searches, pathway and interpro-domain analysis, expression profiling and orthology relation. Comparison of the genomic locations between these genes and sorghum quantitative trait loci (QTLs) showed that 40% of these genes were co-localized with QTLs known for drought tolerance. The genome reannotation conducted using the Program to Assemble Spliced Alignment (PASA), resulted in 9.6% of existing single gene models being updated. In addition, 210 putative novel genes were identified using AUGUSTUS and PASA based analysis on expression dataset. Among these, 50% were single exonic, 69.5% represented drought responsive and 5.7% were complete gene structure models. Analysis of biochemical metabolism revealed 14 metabolic pathways that are related to drought tolerance and also had a strong biological network, among categories of genes involved. Identification of these pathways, signifies the interplay of biochemical reactions that make up the metabolic network, constituting fundamental interface for sorghum defence mechanism against drought stress.

**Conclusions:**

This study suggests untapped natural variability in sorghum that could be used for developing drought tolerance. The data presented here, may be regarded as an initial reference point in functional and comparative genomics in the Gramineae family.

**Electronic supplementary material:**

The online version of this article (10.1186/s12863-017-0584-5) contains supplementary material, which is available to authorized users.

## Background

Sorghum (*Sorghum bicolor* (L.) Moench) is one of the few crops that is able to grow and become productive under dry and more extreme conditions. Several studies indicate that such a unique adaptation of sorghum to arid and semi-arid conditions may be attributed to its recent C4 photosynthetic pathway evolution [1], anatomical structure and physio-biochemical processes [2]. Previous studies have investigated various aspects of sorghum performance using traditional and indigenous knowledge [3, 4], conventional breeding systems that include diversity assessment and resource allocation, molecular breeding and quantitative trait loci (QTLs) mapping [5–7]. Other methodologies include whole genome sequencing, genome scanning, comparative genomics and transcriptomics to describe the biological mechanisms and functional information so as to identify and understand the functional basis of sorghum inherited traits [8–10].

All findings using the above methodologies suggest that there is relatively limited work that has been reported on candidate gene identification for drought tolerance in sorghum as compared to most studied plants such as Arabidopsis [11], Maize [12] and Rice [13]. Sorghum is known for its high genetic variability, however the genes that play rate limiting roles in pathways controlling drought tolerance are not known. For example, approximately 50% of the 34,211 existing protein coding genes lack experimentally validated information and 14% of the sorghum transcriptome (sorghum_79_annotation) represent unknown protein function [8]. Assigning drought tolerance phenotype to any of these genes is apparently not just important for plant transformation to improve sorghum drought tolerance and yield stability but also for marker-assisted breeding, especially in a non-genetically modified crops.

Traditionally, the candidate gene approach aims at a single gene in a pathway in order to measure its tolerance contribution but without a detailed analysis and identification of many and possibly all components of the complex biological processes [14]. However, this approach has been proven to be powerful and potentially effective method for identifying genetic architecture of complex traits, when integrated with in silico analysis [15]. An Integrated In Silico Candidate Gene Approach allows for mapping expression data to metabolic pathways, Interpro-domain analysis, gene expression profiling and analysis of orthology groups to investigate genes of interest by considering functional features of the traits.

The advent of next generation sequencing technologies has accelerated the identification of genes and complex traits for drought tolerance in sorghum, complementing the use of unique genetic resources such as near-isogenic lines, which were commonly used in the past decade to identify complex quantitative traits [7]. However, genomic data sets such as a normalized library of drought-regulated expressed sequence tags (DRESTs) also provide a well-defined view of the transcriptome [16], the so called ‘UniGenes’ that represent putative unique genes. The UniGene database represents a collection of non-redundant stage-wise clustered and unified view of transcriptome that comprise expressed sequence tags (ESTs) that are derived from differentially expressed cDNA libraries [16]. Presently, the sorghum gene space is represented by about 14,000 UniGene clusters in more than 90 diverse libraries from several genotypes [17]. Therefore, the UniGene transcripts expressed under drought conditions, together with their genomic locations represent a collection of candidate genes for drought tolerance.

Furthermore, the present study relies on an updated genome annotation, a dynamic process of gaining additional information on molecular and genome biology. Compared to the rice genome which was annotated 7 times to date [18] and the arabidopsis genome that has been annotated 5 times [19], the sorghum genome has undergone 3 versions of annotation updates since 2009 [20]. To our knowledge relatively few studies have reported on sorghum functional annotation using RNAseq technology [9] or on whole genome sequencing [10]. This work also provides a method for identifying putative novel genes associated with economically important traits whereby two approaches, an intrinsic, that basically relies on a target genomic sequence and extrinsic, that uses external expression and transcriptional evidence, were employed. The current method used in our gene prediction pipeline is a combination of both approaches that serve as a validation protocol [21].

In this investigation, we embarked on an integrated-genomic approach to identify, characterize and prioritize sorghum candidate genes for drought tolerance. We set out to identify drought tolerant genes in the current sorghum annotation by mapping UniGene data obtained from drought resistant libraries. The sorghum genome was reannotated using publicly available experimental data. This study presents a unique approach that complements existing efforts in sorghum research and contributes greatly to further understanding sorghum genomics and comparative studies.

## Results

### Reannotation of sorghum drought responsive genes

**Fig. 1:**
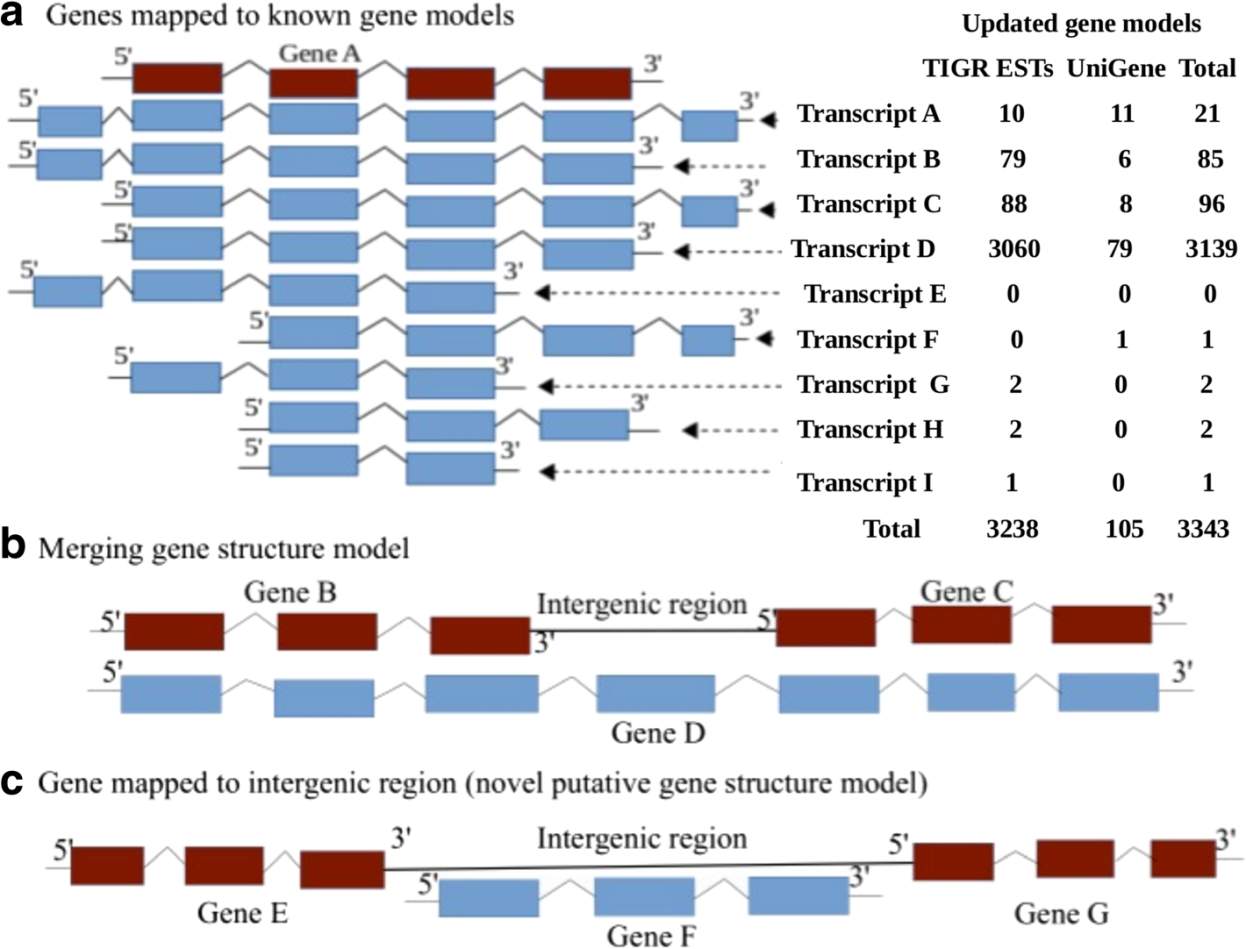
Schematic gene structure model for annotation comparison. In this figure, there are three representations of gene structure models. **a** represents hypothetical map of transcripts to the existing gene model (EGM): ‘Gene A’ denotes a hypothetical EGM to which all transcripts overlapped showing a specific type of updated gene model. Transcript A, B and C each represents an extended overlapping gene at both 5′ and 3′ edges, at only 5′ edge but sharing start position at 3′ edge and at only 3′ edge but sharing start position at 5′ edge respectively. Transcript D represents perfect overlapping gene that conform or share start position at 5′ and stop at 3′ edges. Transcript E and F represent partial overlapping at one edge and extension at another where the former partially overlapped at 3′ and extended at 5′ edge and the latter with an exact opposite pattern. Transcript G and H each denotes a partial overlapping gene that shares start position at 5′ edge and at 3′ edge respectively. Transcript I represents an inner overlapping gene. The values given corresponding to each overlapping transcript in **a** describe the actual number of modified genes in our finding based on TIGR DRESTs and UniGene datasets. **b** represents cross-genic overlapping (merged gene structure model) where two separate EGMs, ‘Gene B’ and ‘Gene C’ were assumed to be merged into a single gene model, ‘Gene D’. **c** represents an illustration of a NGSM ‘Gene F’ that mapped to an intergenic region between the two EGMs ‘Gene E’ and ‘Gene G’ that represent the left and right nearest neighbouring genes respectively. The gene names denote arbitrary example. Each bar represents exon structure and the inverted ‘v’ shaped structure positioned between any two adjacent bars represents intron splicing. The gene model structure with red bars denote EGMs and those with blue are assumed to represent the currently identified genes that mapped to EGMs (transcript A-I), merged gene (‘Gene D’) and NGSM (‘Gene F’). This schematic gene structure model assumes both strand orientations based on the pattern of loci overlapping observed in our results

Sorghum genome annotation was improved by the Program to Assemble Spliced Alignment (PASA) pipeline. Merged genes and transcripts, different isoforms, novel exons and UTRs were identified highlighting annotation update (Table 1; Additional file 1). UniGene data and The Institute for Genomic Research (TIGR) mRNAs obtained from drought responsive libraries were used to reannotate the sorghum genome. Among a total of UniGene clusters, 41 comprised ESTs which were derived exclusively from drought resistant libraries, 24% possessed mixed content libraries and the remaining were without any EST that originates from the same libraries. Drought responsive UniGene clusters were mapped to a total of 123 existing sorghum genes for which no previous report on drought response annotation exists. In addition, 210 gene models were generated which were not previously annotated in the sorghum genome (V1.0, V2.1 and v3.1). A total of 146 of these new genes were drought responsive. Mapping of sorghum UniGene data and TIGR ESTs to the sorghum genome resulted in extensions to the existing gene models (Fig. 1). The gene structure models for a total of 3343 genes (9.6%) were re-defined using the sorghum mRNA data and this included 59 new exons, 72 putative 5’ UTRs and 3499 putative 3’ UTRs (Table 1; Additional file 1; Additional file 2: Table S4). Two genes on chromosome 4 (‘Sb04g008510’ and ‘Sb04g008530’) were merged to form one gene, ‘Sb04g008510_Sb04g008530’ (chr4: 9,869,026–9,888,743). In addition, a novel transcript, Sb04g007110.2.1 (chr4: 7,175,432–7,182,182) was identified and the other two transcripts ‘Sb04g007110.2′ and ‘Sb04g007110.3′ of the same gene ‘Sb04g007110’ were found to be valid single gene model updates. Additionally, we identified 136 alternative splicing events which indicate a source of genomic variation in sorghum for which retained introns and skipped exons contributed 20 and 7% respectively and alternate acceptor and alternate donor accounted for 31 and 10% splice junction respectively. Alternate exon, ends in intron and starts in intron, each contributed 12%, 7% and 13% splice events respectively (Additional file 2: Table S8, S9 and S10). A detailed description of the representative modified gene structure models is given in Fig. 1.

**Table 1:** Description of novel features based on annotation comparison and identified novel gene structure models (NGSMs) based on extrinsic data

Source data	Novel features based on annotation comparison							Identified novel genes		
	Input	3’ UTR	5’ UTR	Exon	^a^Transcript		^b^Genes merged			
					Merged	Novel		Hints		Genes
UniGene clusters	10,619	76	34	33	–	–	–	BCUCs	856	64
TIGR transcripts	20,199	3423	37	26	2	1	2	BCORFs	500	122
								ICGBs	520	24
Total	30,818	3499	71	59	2	1	2		1876	210

Key to legend: ^a^Unique total merged and novel transcripts; ^b^Unique total merged genes; Best candidate UniGene Clusters (BCUCs); Best Candidate Open Reading Frames (BCORFs); Initial Comprehensive Gene Builds (ICGBs)

### Novel gene structure model prediction

**Fig. 2:**
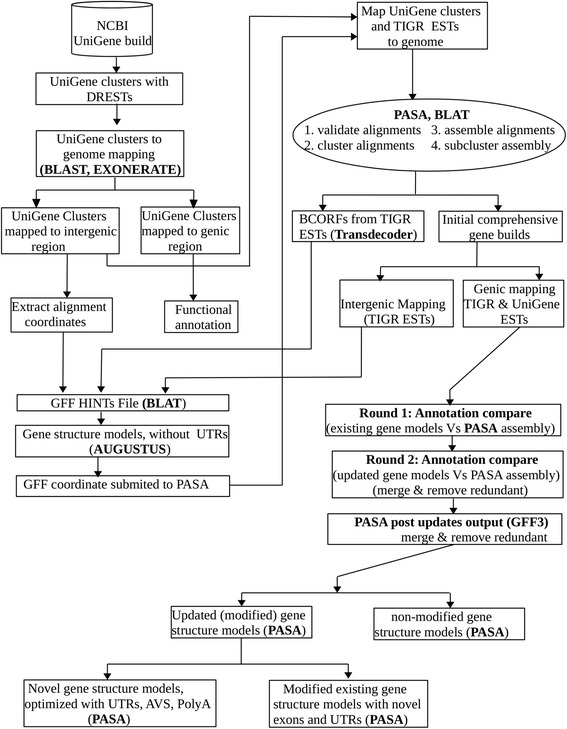
Pipeline for mapping experimental data to reference genome and annotation comparison. This pipeline represents a work flow for identifying known and novel candidate drought responsive genes (CDRGs) and for finding out annotation updates. Identified known putatively uncharacterised genes were functionally annotated. The UniGenes that mapped to integenic region were used by BLAT to generate HINTs and then by AUGUSTUS to identify novel genes which were further optimized by PASA. The PASA pipeline was initiated afresh by cleaning up of any existing output in the MYSQL database using utility codes. The process for annotation comparison was then started by running alignment assembly and by employing the minimum criteria for overlapping transcript alignments and for sub clustering into gene structure (Table 4). Mapping valid alignment assemblies to genome resulted established ICGBs. While the gene builds mapped to the intergenic region that come from the TIGR transcripts were used by BLAT to generate additional HINTs, those mapped to the genic region were used for further annotation comparison. A two round approach was implemented by PASA for processing a complete annotation comparisons: 1st, compared existing gene structure annotations with alignment assemblies and 2nd, re-run, using the output from the first round to capture a few more updates or to verify the initial updates if there was no further updates from the second round. Analysis of alternative spliced alignments and identification of BCORFs were also included in the process. The BCORFs originated from TIGR ESTs were another input to generate HINTs

Novel gene structure models were built based on evidences from 3 initial gene sets, which were all mapped to an intergenic region (Additional file 2, Table S3; Table 1; Additional file 3). A series of alignment steps were carried out to generate HINTs using EXONERATE [22] and BLAT [23] and to build the gene models using AUGUSTUS [24]. We initially identified 414 novel genes which were optimized by PASA [25] (Fig. 2). The gene models were then subjected to a series of screening procedures whereby 210 novel genes were retained. The screening criteria were well proven to filter valid gene structure models such that; 1) the genomic coordinates of the NGSMs were not overlapped even partially, with the coordinate of the existing genes. This was considered primarily as a mandatory criterion for the novelty of the predicted genes, which was also applied if the two genes were predicted in close proximity. Where this was not satisfied, the genes were immediately ignored without looking into additional factor; 2) the lengths of all the predicted genes were considered to be greater than 200 bps and those that did not meet this criterion were also disregarded, even though the first criterion was met. One hundred and forty nine genes were identified where the length of each was greater than 500 bps, of which 68.5% were longer than 1000 bps; 3) the score of the predicted genes, which was the confidence score output by the gene predictor itself, was set to be a minimum of 0.5 of (0–1) for the genes to be valid; 4) the percentage evidence support, where prediction was based on homology, was considered to be more than 50 of which the majority displayed 100% (Additional file 1); 5) strand orientation of the predicted genes in relation to the existing genes or the currently predicted genes if they were neighbours, was considered important. The intergenic distance was mostly considered valid with a minimum of 100 bases, which was very important to enhance the validity and novelty of the NGSMs (Additional file 2: Figure S3). It was not necessary to consider these parameters in order of their weight, but they all contributed to the valid results. However, for the novelty of the genes and accurate prediction, we considered the first two criteria to be mandatory. The genomic coordinates for NGSMs predicted by AUGUSTUS and then optimized by PASA pipeline programs were compared to known sorghum genes coordinates (Sbi1.4, v2.1 and v3.1, latest release). Genes which satisfied any of the 4 listed criteria were considered valid and all that didn’t satisfy this stringency were disregarded.

Of all the predicted novel genes, 12 were complete gene structure models (having both 3′ and 5’ UTRs), 15 genes were with 3’ UTR only and 2 genes were identified with 5’ UTRs only (Additional file 2: Table S5; Fig. 2; Additional file 3). This means that 29 genes had at least 1 UTR edge (semi-complete gene structure at 3’ UTR edge only, or at 5’ UTR only or both) and the remaining were partial models without any UTR segment but with the start and stop codons (Additional file 2: Table S5; Fig. 2; Additional file 3). The total number of novel genes that accounted for drought response represents 69.5% (Additional file 1; Additional file 2: Table S5). While 112 genes (53.3%) had extrinsic evidence for which percent evidence support was recorded based on sequence homology, the other 46.6% were predicted based on ab-initio*,* using intrinsic data. Additional file 1 contains a complete list of novel genes identified in this study with description of the gene models annotation.

### Intronless (single exonic) novel genes

A total of 105 novel genes among the 210, were single exon genes of which 74 represented drought responsive (Additional file 1). Among these, 2 single exonic intronless genes exhibited a complete gene structure, 4 were partially complete of which 1 is 5’ UTR and the other 3 retained 3’ UTR, whereas the remaining 99 were truncated (Table 2; Additional file 1). Since there is some positive correlation between intron loss and processed pseudogene and truncation as a common feature between the two events [26], we speculate that some of the identified single exonic genes are pseudogenes based on the criteria set by Ensembl (Ensembl Gene Set) [27]. The pattern and distribution of exons and introns for the novel genes throughout the sorghum genome is shown in (Table 2) and the pattern of their number and average length is given in **(**Additional file 2: Figure S2; Additional file 2: Table S11).

**Table 2:** Description of exons and introns distributions for the novel genes throughout genome

Scaffolds	Total features per scaffold			Exons per gene		Length (bp) of features										Scaffold size
						Shortest			Longest			Total		Average		
	Exons	Introns	Genes	Max^a^	Ave^b^	Exons	Introns	Genes	Exons	Introns	Genes	Exons	Introns	Exons	introns	
Chr1	66	38	28	9	2.4	20	77	209	2065	3614	8778	23,780	23,480	360.3	617.9	2164–67,845,075
Chr2	45	25	20	7	1.8	6	69	254	1283	1146	4690	13,378	4853	297.3	194.12	135,092–64,307,455
Chr3	35	14	21	4	1.6	3	103	236	2297	3392	3689	15,921	11,234	454.8	802.4	25,612–73,118,483
Chr4	60	37	23	9	2.6	7	66	224	2732	10,968	13,188	20,569	33,993	342.8	918.7	10,238–67,290,539
Chr5	44	25	19	5	2.3	19	71	245	2018	6695	8795	12,688	25,038	288.4	1001.5	43,486–33,757,957
Chr6	23	12	12	5	1.9	31	76	293	1337	1060	6502	7588	4018	329.9	334.8	11,607–52,987,788
Chr7	39	18	20	4	2	6	79	224	3156	2505	7905	18,860	9241	483.6	513.4	41,803–56,863,519
Chr8	45	20	25	5	1.8	6	71	218	2858	2680	4372	25,730	15,539	571.8	777	10,323–34,152,034
Chr9	44	24	20	6	2.2	44	69	233	3614	15,338	16,668	24,142	27,308	548.7	1137.8	17,759–55,481,927
Chr10	38	18	20	5	1.9	24	72	212	1855	6768	13,475	16,358	17,936	430.5	996.4	13,087–53,827,720
Super	2	0	2	2	1	739	0	739	887	0	889	5695	0	798.2	0	4–8,720,612
Ava	40	21	19	6	2.1	81	67	275	2446	5580	8141	17,958	19,777	479.1	735.4	28,289–51,668,465

Key to legend: ^a^ Maximum; ^b^Average. This data depicts that the least number of novel genes (2, 1%) were identified in super scaffold, probably owing to its relative smaller size and lower gene density [8] and that highest prediction was from chromosome 1 with 28 genes showing its biggest size

### Analysis of protein-protein search and protein domain

Among the 210 predicted novel genes, 146 were drought responsive for which protein-protein search against non-redundant protein database using blastP were conducted. We identified that 60% of the query proteins were mapped to the known proteins database of which 35% received ≥80% identity. The rest (40%) remained unmapped (Additional file 4). On the other hand, analysis of pfam revealed 32 different protein domain and families to which one or multiple protein sequences of the predicted drought responsive genes (DRGs) were mapped. Of these, 71.9% were identified to have clan annotation suggesting the presence of multiple lines in protein domain, while the rest were devoid of any clan representation and were annotated with a single line. The descriptions for blastp and pfam analysis are shown in Additional file 4 and Additional file 5 respectively.

### Metabolic pathways analysis

**Fig. 3:**
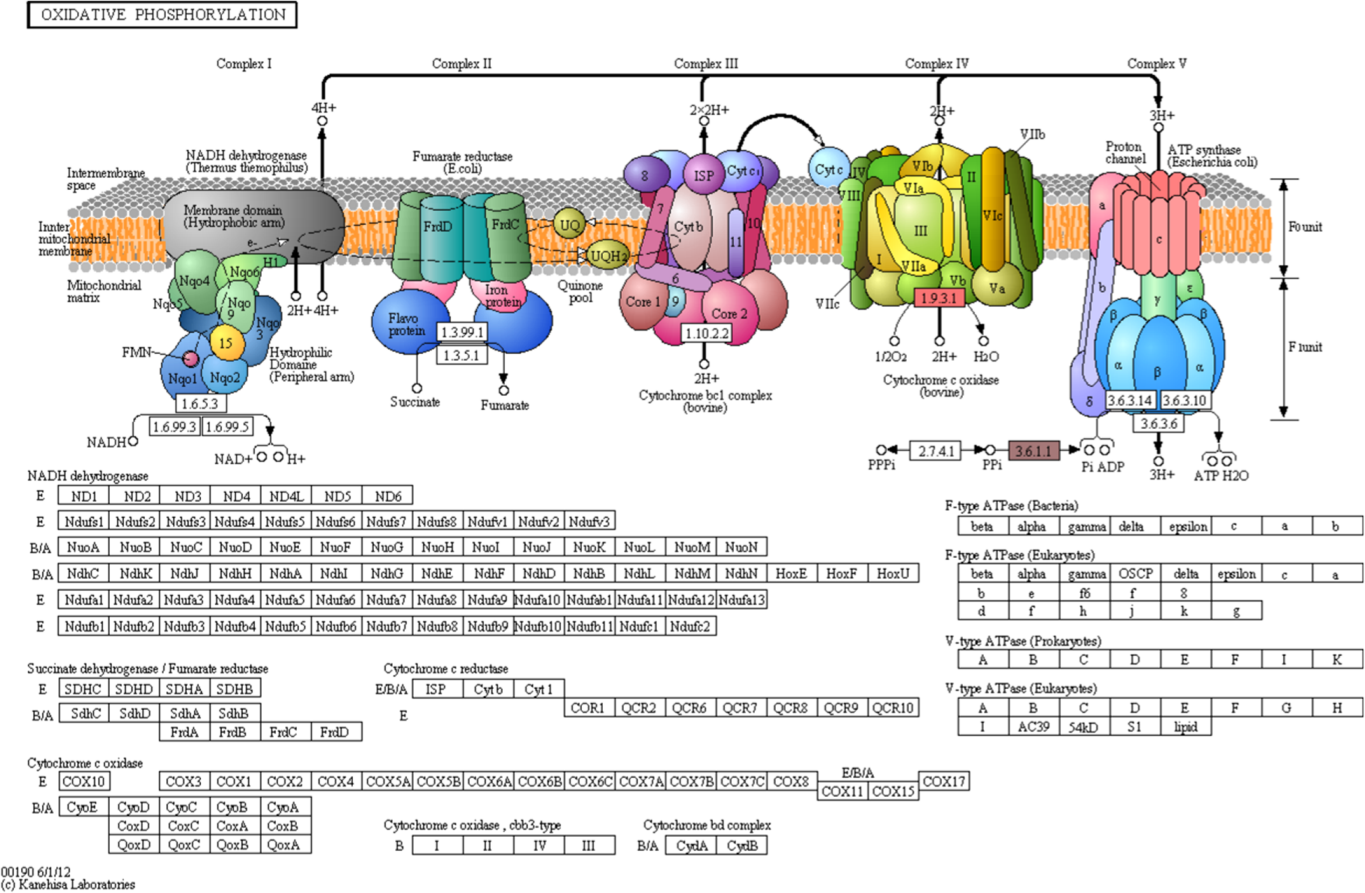
Oxidative phosphorelation metabolic pathway. This represents one of the 14 metabolic pathways identified in this study and is associated with the production of respiratory energy in mitochondria, a power house of the cell. Cytochrome c oxidase subunit 1 (EC: 1.9.3.1; Additional file 6), the enzyme encoded by sorghum gene cox1 was identified to be involved in the catalytic reaction of the final step of protein complex (complex IV) in the electron transport chain. In addition, inorganic diphosphatase (EC: 3.6.1.1; Additional file 6) was identified to be involved in the electron transport system by catalysing the conversion of diphosphate into monophosphate. This enzyme controls the amount of inorganic phosphate (Pi) that should be coupled with adenosine dinucleotide phosphate (ADP) in the last step of oxidative phosphorylation, a phenomenon thought to be involved in counteracting an imbalance of reactive oxygen species caused by drought stress

A total of 14 Kyoto Encyclopaedia of Genes and Genomes (KEGG) pathways were mapped to the 123 drought responsive UniGene clusters. Twelve of these metabolic pathways contain enzymes encoded by sorghum genes (Additional file 6). The other 2 namely drug metabolism-other enzymes and purine metabolism are catalysed by cholinesterase (EC:3.1.1.1) and adenylpyrophosphatase (EC: 3.6.1.3) respectively for which we did not find any encoding gene currently annotated in the sorghum genome. We thus, suspect that these are novel pathways for sorghum. We arbitrarily selected five metabolic pathways (Additional file 2: Figure S5-S11) to discuss the results in detail. A detailed description of all the pathways and a total of 32 genes identified and functionally enriched are indicated as the potential drought responsive candidates (Additional 1: Table S15; Additional file 2: Figure S5-S11). Of the other KEGG pathways identified, oxidative phosphorylation is indicated in Fig. 3.

Glucosinolate biosynthesis in sorghum is associated with dhurrin (cyanogenic glucosides) synthesis for which the gene CYP79A1 [EC:1.14.13.41] is responsible to catalyse the chemical reaction. This finding shows that there is a likely integrative metabolic role played by the 3 pathways namely Pantothenate and CoA biosynthesis (PCAB) (EC:2.6.1.42), Valine, Leucine, and Isoleucine biosynthesis (VLIB) (EC 2.6.1.42) and Valine, Leucine, and Isoleucine degradation (VLID) (EC 2.6.1.42) which are coordinated by 3 peculiar genes (‘Sb04g010240’, ‘Sb06g025140’ and ‘Sb09g008180’). These genes encode a common enzyme called branched-chain amino acid transaminase (EC 2.6.1.42) that is responsible for the amination of the 4 methyl-2 oxopentanoate. Altogether, 28 genes were identified to be involved in this 3 pathways among which are the 3 aforementioned ones.

A metabolic pathway, oxidative phosphorelation is known to be involved in the production of energy by maintaining mitochondrial respiration at times of water stress condition [28]. In sorghum, 2 genes namely COX1 and ‘Sb09g022400’ were identified to be involved in the oxidative phosphorylation and responsible for encoding cytochrom c oxidase 1 and diphosphatase respectively, both of which take part in the electron transport system (Fig. 3).

### Functional gene ontology (GO) enrichment analysis of the genes involved in the pathways

**Fig. 4:**
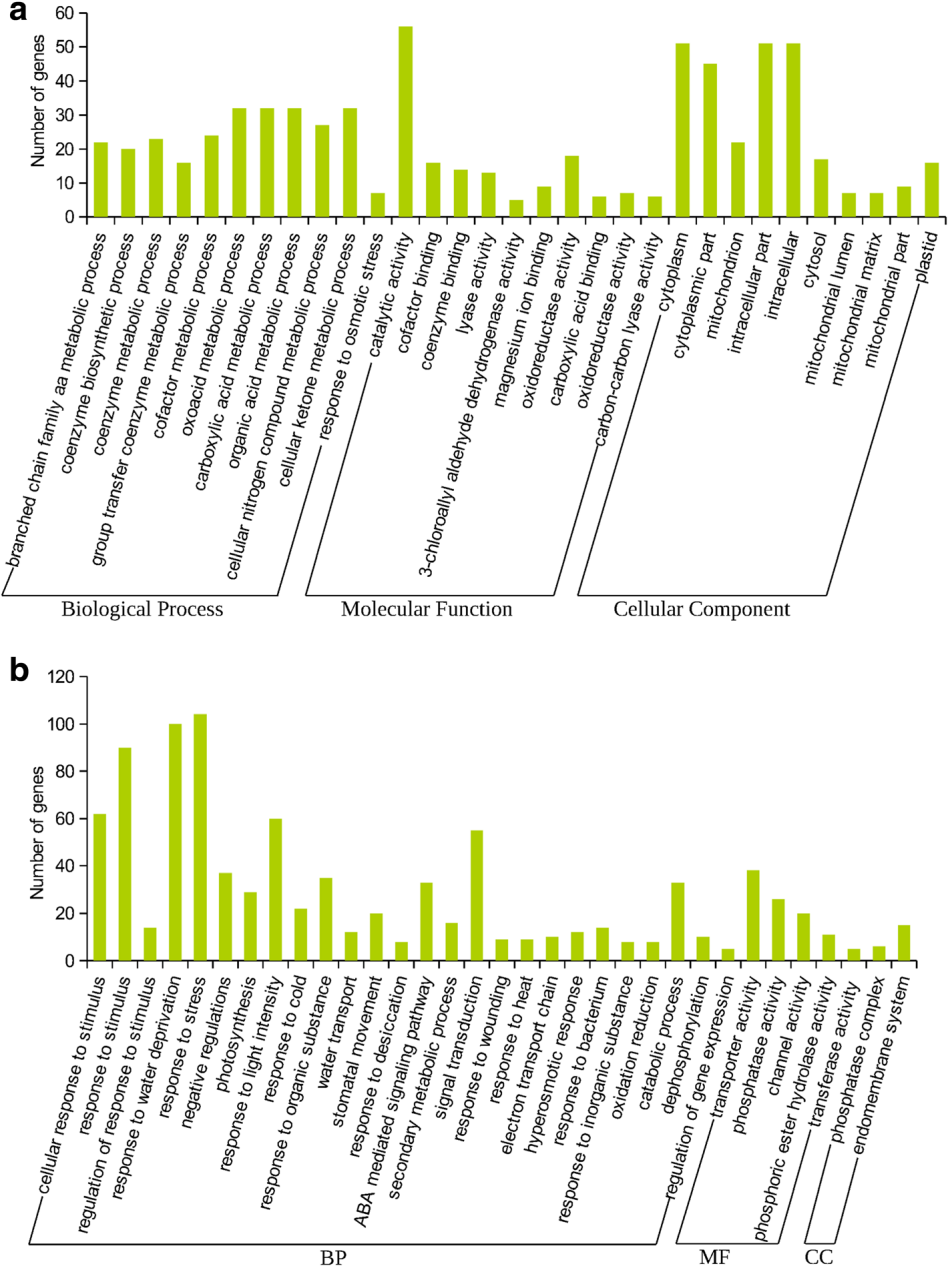
Representation of the GO classification. Gene Ontology terms assigned to the drought responsive sorghum UniGene clusters that encode genes involved in the drought related pathways based on the blast hit obtained against the non-redundant database are classified into three main categories namely BP, MF and CC and 31 subcategories (**a**). Likewise, the enriched GO-terms from the differentially expressed (up and down-regulated; *p*-value <0.05) sorghum genes and orthologs that were queried based on the high-score blast hit against the non-redundant database are classified into three main categories as mentioned above and 33 subcategories (**b**). While the left y-axis represents the number of genes associated with the subcategories, the x-axis indicates the specific subcategory involved in the main category

A total of 477 sorghum genes in all the pathways were identified to which 583 significantly enriched GO-terms were assigned (*P*-value; False Discovery Rate (FDR) < 0.01). However, analysis revealed that only 32 genes (6.7%) were responsible for encoding enzymes that catalyse substrate conversions in the respective pathways (Additional file 6; Additional file 2: Table S15). The assignment of GO terms to each UniGene cluster involved in the pathway analysis, represents the functional categorization of the specific UniGene cluster and the corresponding sorghum genes. Based on the GO classification, a total of 31 subcategories were distributed into the 3 main GO categories such that 11 subcategories were assigned to the biological process (BP), 10 subcategories to the molecular function (MF) and another 10 to the cellular component (CC) (Fig. 4a). UniGene clusters that accounted for 82% of the genes in the category of BP were mainly involved in the metabolic processes such as oxoacid metabolic process (GO:0043436), carboxylic acid metabolic process (GO:0019752), organic acid metabolic process (GO:0006082) and cellular ketone metabolic process (GO:0042180). On the other hand, the response to osmotic stress had relatively less representation of UniGene clusters (Fig. 4a). The assignment of GO-terms to UniGene sequences in the MF was relatively lower when compared to the other 2 main categories. Here, catalytic activity (GO:0009651; 37.3%) was found to be dominant followed by oxidoreductase activity (GO:0016829; 12%) and co-factor binding (GO:0006725; 10.7%). On the contrary, the category CC contributed for the annotation of a total of 276 genes which were linked to drought responsive UniGene clusters to which enriched GO-terms from 10 subcategories were assigned. Of these subcategories, cytoplasm (GO:0016835), intracellular (GO:0044444) and intracellular part (GO:0005737) were included each with UniGene clusters that accounted for 18.5% of the total number of genes involved in the CC.

### Interpro-domain analysis

Interpro-domain analysis clearly shows that the frequency of protein domains in the sequences varies greatly. Protein domains represented 33% of the main categories of interpro-domains identified. A total of 630 interpro-domains were identified of which the known signature represented 60.5% (Additional file 2: Figure S12). Table 3 shows description of the top ten interpro-domains in decreasing order of frequency among the total with the known signature domains.

**Table 3:** Description of the top ten interpro-domains in decreasing order of frequency

Interpro-domain	Accession^a^	F, P^b^	Functional description	References
DnaJ domain	IPR001623	22, 6.4	Acts as protein chaperon; cooperation of Hsp40 with Hsp70 and endosomal trafficking^c^	[58, 59]
Gamma thionin	IPR008176	18, 5.3	Plant defensins induced in response to drought	[82]
Ribosomal protein L29e	IPR002673	17, 5	Forms part of the 60S ribosomal subunit, structural constituent of ribosome^d^	[83]
Zinc finger, CCHC-type	IPR001878	17, 5	Drought stress response in plants	[57]
DUF4281^e^	IPR025461	16, 4.7	Protein domain functionally uncharacterised, found both in prokaryotes and eukaryotes	[84]
RNA recognition^f^	IPR000504	16, 4.7	Expression of EgRBP42 transcript under drought stress	[85]
Cytochrome c oxidase, subunit VIIa	IPR003177	14, 4.1	Catalyses the reduction of oxygen to water in the inner mitochondrial membrane forming the functional core of the enzyme complex^g^	[86]
Oligopeptide transporter family^h^	IPR000109	14, 4.1	Showing an enhanced response in 35S:ABF3 plants that may contributing to drought-tolerance	[87]
Peptidase S10, serine carboxypeptidase	IPR018202	13, 3.8	Protein recognition and binding, serine carboxypeptidase-like gene OsBISCPL1 in rice is involved in regulation of defence responses	[88]
CBS domain	IPR000644	12, 3.5	Transcript levels of CBS domain containing proteins are altered in response to drought	[89]

Key to legend: ^a^Interpro accession; ^b^Frequency of occurrence, %; ^c^Intracellular; ^d^involve in translation and ribosome biogenesis; ^e^Protein length range between 147 and 232 amino acids with known two functionally important conserved residues (W and P); ^f^motif domain; ^g^transferring the electrons from cytochrome c via its binuclear copper A centre to the bimetallic centre of the catalytic subunit 1; ^h^Proton-dependent

### Analysis of gene-expression profiling

**Fig. 5:**
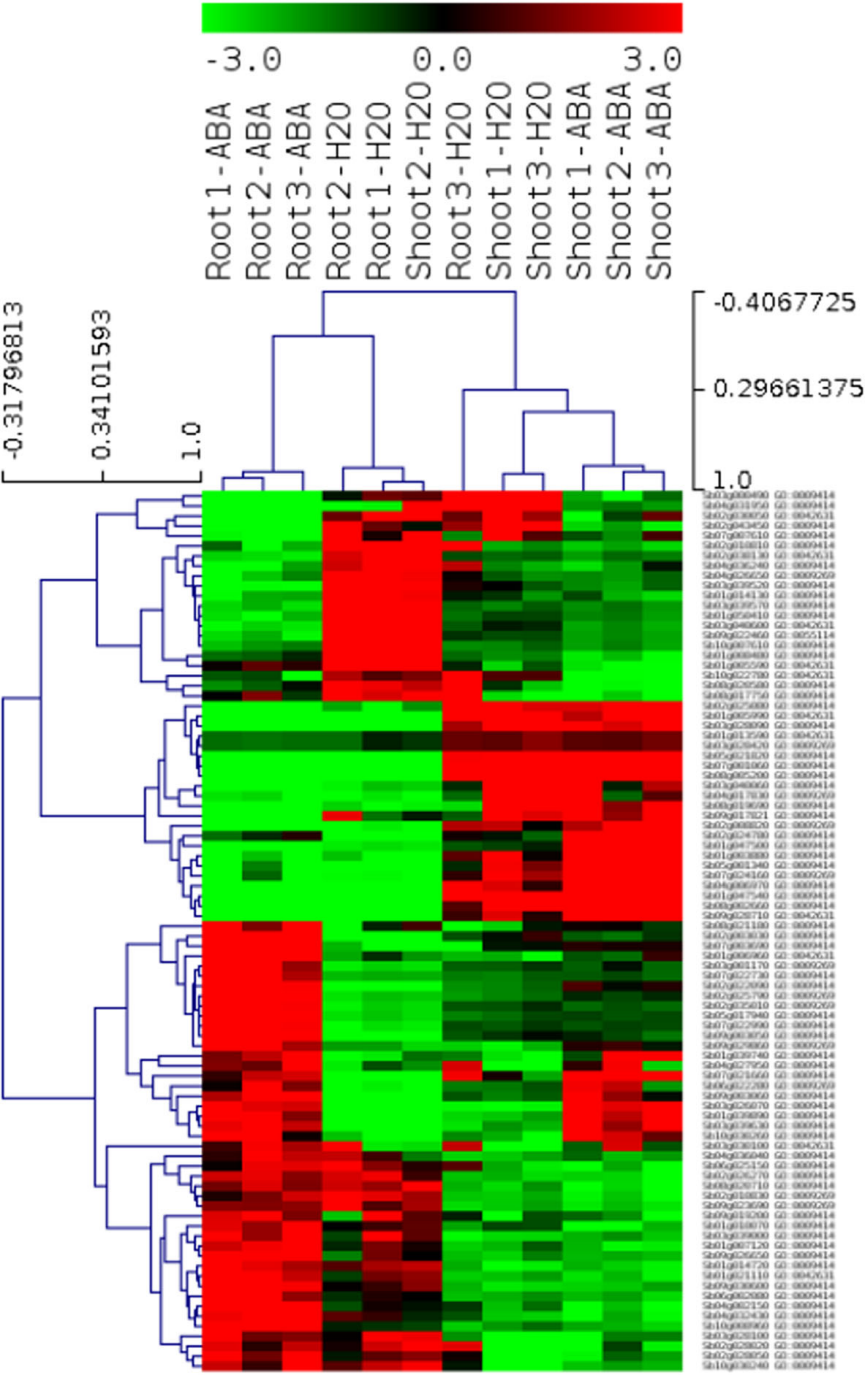
Heat-map showing differential gene expression based on sorghum RNA-seq dataset. The hierarchical clustering of gene expression profiling in this figure is associated with the information derived from the sorghum drought related ontology terms and the gene expression omnibus (GEO) database. The figure shows heat map depicting up and down-regulated genes under drought condition based on data from sorghum RNA-seq in response to osmotic and abscisic acid stresses. The rows represent the genes, while the columns represent the biological samples. The red color denotes the up-regulation, while the green shows down-regulation of the genes

Based on the analysis of expression data from sorghum, 46 significantly expressed genes were shown to have direct association with drought tolerance with tissue -related effects. However, based on the evaluation of the treatment effect only, 42 genes were shown to have significantly up-regulated irrespective of tissue specificity (Fig. 5; Additional file 2: Figure S13; Additional file 7). This shows that the gene expression based on tissue-specificity provided higher representation of drought responsive genes than with the influence of drought stress regardless of specificity in tissue involvement, which is in agreement with the previous work on sorghum stress response [11]. A representation of a significantly up-regulated genes is shown using volcano plots, reflecting the tissue type contributed to the gene expression that is more significant than the treatment effect (Additional file 2: Figure S13; Additional file 7).

**Fig. 6:**
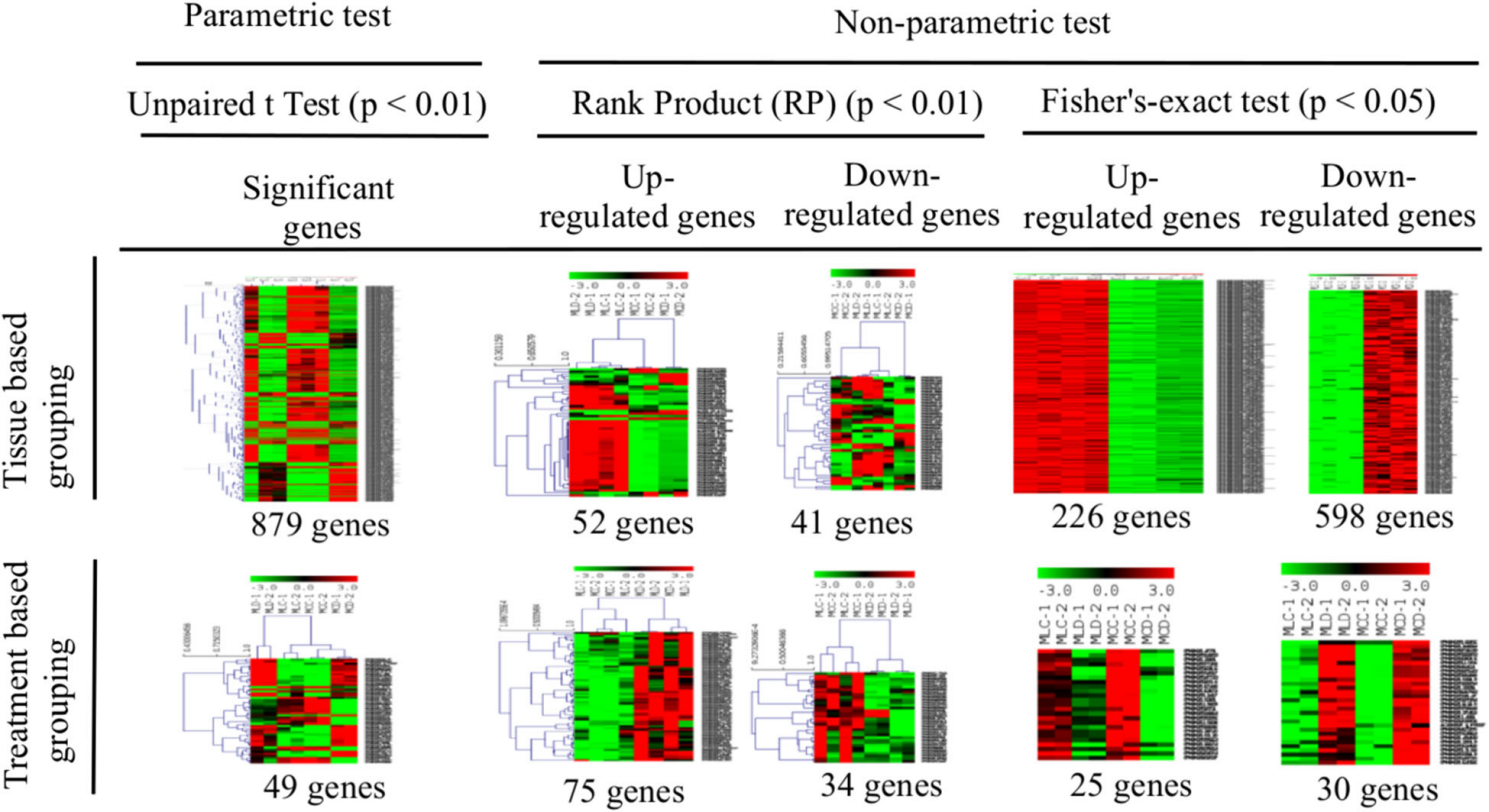
Heat map showing up and down-regulated sorghum orthologs in maize from RNA-seq data. The comparison of gene expression pattern based on parametric (unpaired t-Test or between subject comparison, *p* < 0.01) and non-parametric test (Rank Product (RP), *p* < 0.01), and Fisher’s Exact test (*p* < 0.05) shows the up and down-regulated genes across treatment and tissue based grouping. Evaluation by treatment based grouping was determined to see significant difference in gene expression due to effect of differential condition under which the samples were tested while tissue based grouping was used to detect the effect of differences in tissues on the gene expression. All data showing significant expression, either up or down regulation of genes in both groupings represent results obtained under drought conditions for ovary and leaf meristem tissues

Sorghum orthologs corresponding to a recently published maize RNA seq data [29] were also evaluated (Fig. 6), where a list of tissue specific up and down regulated genes were identified under drought conditions. We used 140 genes out of the list of drought responsive genes identified in their work and queried sorghum orthologs based on the orthologous pairs recorded and identified 54 sorghum genes with >90% identity and high level confidence. Out of these, 53 were annotated for enriched GO-terms associated with drought responses (Additional file 8). Using the raw data from the same study, we applied three independent statistical methods and discovered 45 significantly expressed genes that were not included in the published result [29]. These were subjected to gene enrichment analysis where 12 sorghum orthologs were found functionally enriched for drought response (*P*-value, FDR < 0.05).

The pattern of gene expression for these orthologs was analysed using both parametric (unpaired t-Test, *p* < 0.01) and non-parametric tests (rank product, *P* < 0.01 and Fishers’s exact test, *p* < 0.05). Statistically significantly expressed 49 and 879 genes were identified using unpaired parametric t-Test following treatment and tissue based grouping respectively. On the other hand, 75 and 34 up and down-regulated genes respectively were identified using rank product under drought condition, based on treatment grouping. Tissue based grouping for rank product revealed 52 up regulated and 41 down regulated genes under the same condition. Similarly, using the Fisher’s exact test, 55 genes were over-expressed based on treatment grouping of which 45.5% were up-regulated. Again, using the same statistical test and based on tissue related grouping, 824 genes were identified of which 27.4% were up-regulated. This result demonstrates the comparison of gene expression pattern based on different statistical models showing the up and down-regulated genes for tissue specific drought stress response (Fig. 6). The distribution of significantly expressed genes under drought condition pooled from different statistical models is shown using Venn diagram [30] in Additional file 2: Figure S15.

The combination of these significantly expressed maize expression data originated sorghum orthologs with the genes identified based on sorghum expression profiling, provided 100 non-redandunt genes responsive to drought stress of which 97% were GO annotated and 80 were functionally significantly enriched (Additional file 9).

### Functional GO-enrichment and GO classification based on gene-expression

Based on the sorghum RNA-seq data evaluated, enriched GO-terms representing significantly expressed genes from a total of 33 subcategories were grouped into 3 main categories (Fig. 4b). The category biological process contributed to the largest share of GO annotation by 79% GO-terms to which 87% of genes were assigned. However, the molecular function accounted for the relatively lower classification of GO- terms (15%) to which 11% of the significantly expressed genes were associated. On the other hand, the cellular component category classified 6% enriched GO- terms only, to which 2% of expressed genes were associated (Fig. 4b, Additional file 7). The GO-terms ‘response to stress’ (GO:0006950, 13% genes) and ‘response to water deprivation’ (GO:0009414, 12.3% genes) were the dominant subcategories in the cluster of the main category biological process followed by other 4 subcategories, ‘response to stimulus’ (GO:0050896), ‘cellular response to stimulus’ (GO:0051716), ‘response to light intensity’ (GO:0009644) and ‘signal transduction’ (GO:0007165) to which 11, 7.6, 7.4 and 6.7% of significantly expressed genes were respectively associated. The major subcategories that accounted for the GO annotation in the main categories of molecular function and cellular component were the set of GO-terms in transporter activities that include (GO:0005372, GO:0022803, GO:0022891 and GO:0022892) and endomembrane system (GO:0012505) to which 38 and 71% of the genes were associated respectively. The least dominant subcategory of the GO classification that contributed to the GO annotation were post-transcriptional regulation of gene expression (GO:0010608, 0.6% genes), transferase activity transferring alkyl or aryl groups (GO:0016765, 5% genes) and protein serine/threonine phosphatase complex (GO:0008287, 29% genes) in the category of biological process, molecular function and cellular component respectively (Fig. 4b; Additional file 9).

Fifty three sorghum orthologs identified with GO annotation based on the known maize drought responsive genes [29] were functionally enriched for which 119 drought related GO-terms were identified (*p*-value <0.05; Additional file 8). Based on the maize RNA-seq raw data, on the other hand, 1079 significant non-redundant genes were resulted from the combined analysis of the three statistical tests with 45 significantly expressed genes supported by all the statistical models (Additional file 2: Figure S15; Additional file 8). These up regulated genes held up by all the models were used to query 41 sorghum orthologs (> 90% identity and high level confidence) using ENSEMBL BIOMART [31] from which 32 annotated and 12 functionally enriched genes were obtained (Additional file 2: Figure S16; Additional file 8). This suggests that sorghum genes identified from maize orthologs showed conserved functional similarity in the drought stress response notably in activities related to reproduction, photosynthetic cellular metabolic process and ion and chlorophyll binding typically involving both photosystems I and II. The combined description of GO annotation from the sorghum and maize expression data is shown in Additional file 9.

Based on the GO classification, cellular and metabolic processes that include ‘responses to stimulus’ constitute a major task of significantly enriched genes involved in the biological process. While organelle, cell and cell parts serve as the integral component for the genes assigned to cellular component, ion binding as a sole activity represents the main function of the majority of the genes with only a few that take part in transcriptional regulatory and structural molecular activities (Additional file 2: Figure S16).

### Analysis of orthology groups

**Fig. 7:**
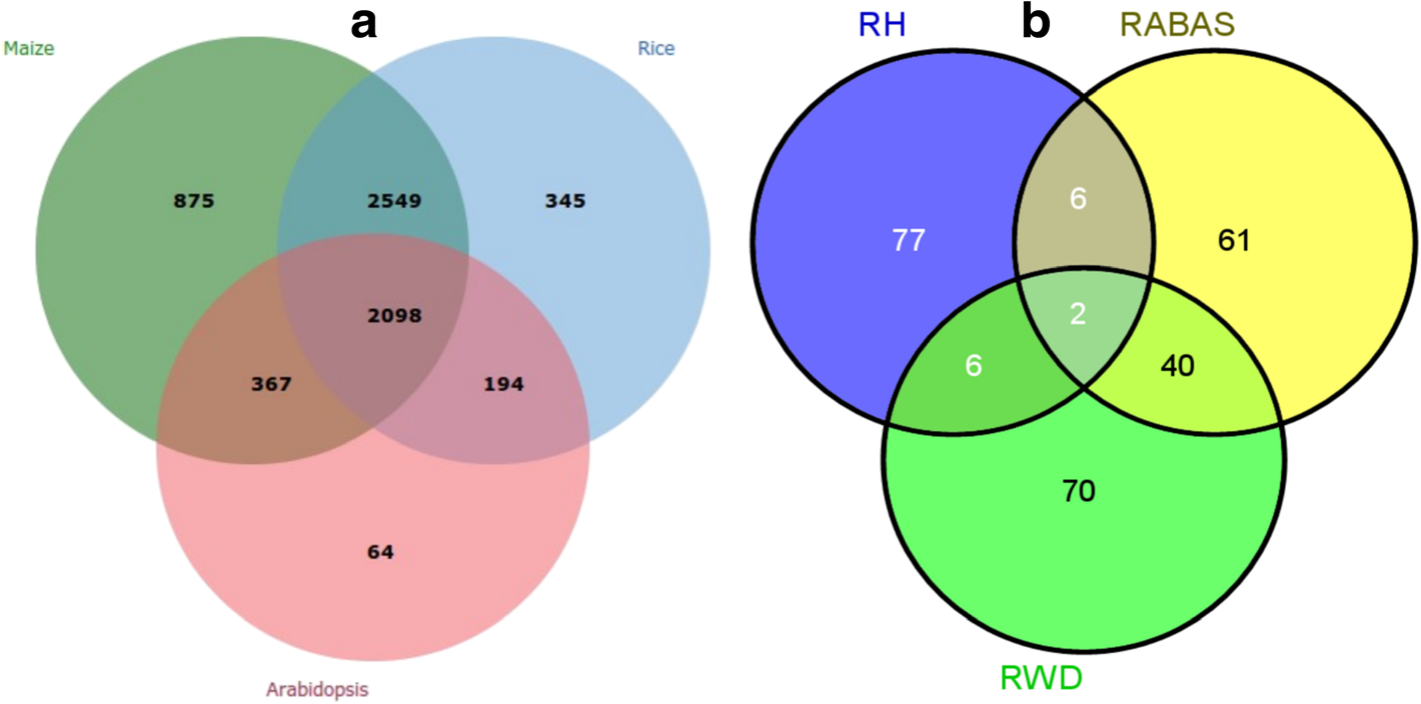
Description of sorghum orthologs across species and drought related GO terms. Key to legend: RWD = response to water deprivation; RH = response to heat and RABAS = response to ABA stimulus. The Venn-diagram shows patterns of shared sorghum orthologous gene clusters among its relative species and GO terms related to drought stress. **a** shows the distribution of shared sorghum orthologs among species, giving some clue on evolutionary implication and functional crosstalk of genes and on the extent of shared conserved syntheny among species related to sorghum. Closely related species (eg. maize and rice) share higher conserved sorghum orthologs (2549 genes) than relatively distantly related species to sorghum, for example maize and arabidopsis only share 367 sorghum orthologous genes and rice and arabidopsis share 194 sorghum orthologs. Surprisingly, 2098 sorghum orthologs shared among all the species seemingly represent ancestral gene families. All the genes in the diagram represent sorghum orthologs in the respective species. The non-shared ones indicate the unique sorghum orthologs found only in the corresponding species. **b** shows the pattern of distribution of genes involved in key selected drought related GO-terms. Functional overlapping was indicated as a clue for gene network among categories involved in complex stress responses with some genes playing a rate limiting role. For example, two genes ‘Sb09g026860.1’ and ‘Sb07g014940.1’ are shared and act in all the pathways. Pathway controlling response to water deprivation shares 40 overlapping genes with the one controlling response to ABA stimulus and six genes with the pathway regulating response to heat (Additional file 2: Table S12). Similarly, the pathway controlling response to ABA stimulus and that controls response to heat share six genes between them. On the other hand, 265 unique sorghum orthologs were identified in total for drought related responses with almost equal proportion of unique genes associated to each of the three Go-terms

Out of the 6915 non redundant orthologs initially identified from the three species related to sorghum, 93% with >50% identity and high confidence level were screened (Additional file 10). Prior to embarking on ontology enrichment using the combination of all the orthologs recovered, we determined to see the extent of species representation and subtotal genes commonly identified by more than one species (Additional file 10 and Fig. 7). To this end, 2098 genes were found to be common in all the species (Fig. 7) and the patterns of sorghum orthologs with respect to the corresponding species was shown based on the input sorghum genes (Additional file 10).

### GO enrichment analysis of genes through orthology groups

The consideration of only genes that are commonly represented in all species in the GO enrichment analysis, did not result in significantly high gene enrichment coverage suggesting that partly the non-common orthologs which potentially contribute to drought tolerance seem to remain unrepresented in the GO enrichment and partly the common genes represent only 30% of the initial total figure and does not seem to be fully representative. It was therefore, necessary to expand the analysis to include all qualifying orthologs. Then, 6321 GO-annotation and 239 significant GO-terms were identified (*p*-value, FDR < 0.05). We reduced the final number to 1102 highly enriched DRGs by selecting a ‘response to stress’ as a key drought associated GO-term and further to 262. Interestingly, significant number of genes validated by GO functional enrichment were identified. This includes genes which were involved in responses to water deprivation (118), desiccation (21), heat (91), ABA stimulus (109) and ABA mediated signalling pathways (37) and which are associated with the corresponding GO terms (Additional file 2: Table S12; Figure S17).

**Fig. 8:**
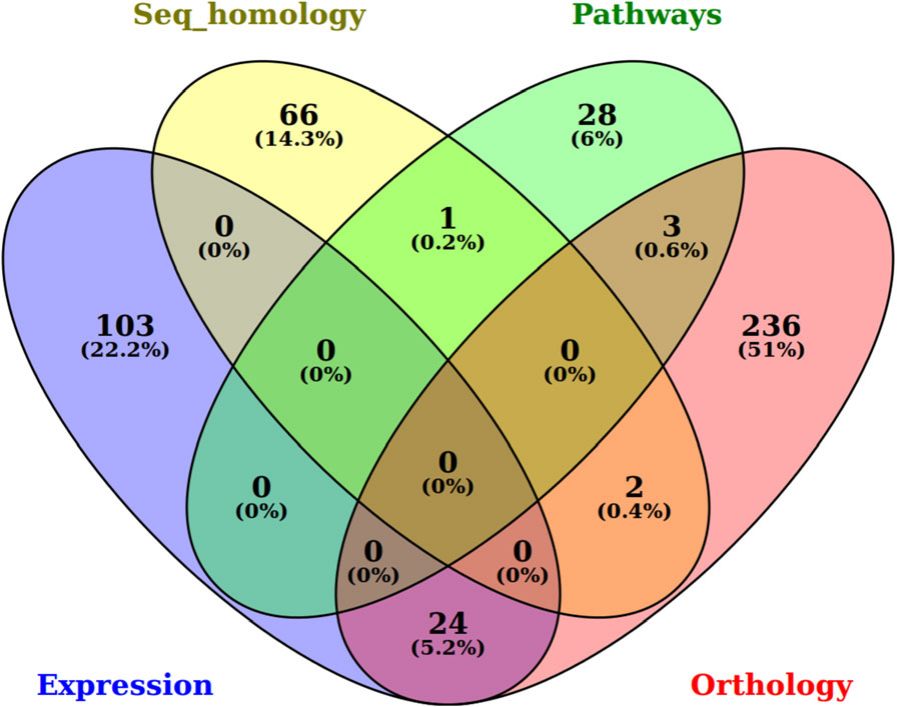
A summarized description of the outputs for the findings of the various analytical approaches. The Venn-diagram shows the number of identified genes and the corresponding percentage in a particular approach used in this study. The numbers in the peripheral regions, parts not overlapped, show unique findings of the particular method, whereas the numbers in the overlapping regions of the circles show the shared values among the methods. This description doesn’t include the results based on genome annotation. Seq_homology, denotes sequence homology

A summarized output of the findings in this study shows that the approach applied to identify and prioritize potential candidate DRGs is reliable. In total, 470 identified non-redundant significantly enriched genes were pooled from all the approaches used (Fig. 8), without including the results that contributed to the update of the genome annotation. While no significant overlap of the results was shown with only 1.2% of the genes identified that shared among the methods, an integrative and comparative approach used to identify the genes that confer drought tolerance suggests the validity of the various sources of independent dataset that were used.

### Identification of target genes associated with different drought QTLs

A total of 187 currently identified DRGs were identified to be associated with different sorghum QTLs known for drought responses. By comparing the genomic coordinates of the target genes with regions harbouring QTLs, it was possible to figure out the regions of co-localization associated with drought tolerance. The identification of these genomic regions does not just indicate the colocalization of the DRGs with the QTLs, but also implicates the functional links of the colocalized genes with stay-green and other known traits such as grain yield, grain weight, flowering time, chlorophyll content, chlorophyll florescence and seed dormancy in sorghum. In this result, fewer single-gene-QTL association was identified as compared to multiple intra-QTL genes that accounted for 94% co-localization (Additional file 11). Among others, 2 HSP70 genes (Sb09g004170 and Sb09g022580) were associated with stay-green QTL (Stg1) that were identified in previous study [32]. Moreover, 36 genes were likely to be associated with QTLs for nodal root angle that are colocalized with drought adaptive traits. Again, 5 QTLs identified for grain yield based on genotyping-by-sequencing markers [33] were co-located with 50 DRGs. While QTLs mapped for flowering time were associated with 20 DRGs, those known for stay-green traits were associated with 52 target genes. In addition, 23 genes were found to be associated with 5 QTLs that are responsible for seed dormancy in sorghum of which qGI-3 and qGI-7 were each co-localized with more than 30% of the genes (Additional file 11).

Target DRGs were also examined for the likely association with the QTLs using sequence alignment approach (Additional file 12). Sequence alignment of selected DRGs with the fine mapping of a major QTL (qGW) for grain weight in sorghum [34], provided 22 genes that received the best hit with e-value <1e-100 and percent identity >80, among which was Apetala 2 (AP2), a plant specific drought inducible transcription factor gene (Sb02g025080).

## Discussion

The detection of genetic determinants of complex traits on an integrated in silico basis, as it was determined in this study, seems to be the best approach to identify candidate genes for drought tolerance. Mapping data to the reference genome is not just important for molecular characterization of genome structure and evolution in the grass family [35], but also vital for comparative genomics in aspects including but not limited to predicting and verifying gene models, identifying and characterizing putative known genes, improving genome annotation, and identifying homologs between genomes of related species in the eukaryotes [36]. Sequence similarity search now for more than two decades since the introduction of BLAST [37] has been the focus in DNA or protein query search against known databases with likelihood of matched sequences on similarity measure returning a set of high-scoring alignment pairs (HSPs) and reflecting evolutionary relationship. In this study, an integrated and comparative in silico approach generated a wide array of CDRGs in sorghum. Mapping UniGene clusters to sorghum genome captured 123 DRGs not ascribed in sorghum EGMs but only classified as either hypothetical, putative uncharacterised or unknown proteins. Because UniGene clusters constituted new drought expressed ESTs which represent a useful approach for gene identification, this finding provides improvements to the sorghum genome functional annotation. Applicability of the method that utilized expression data from 92 different sorghum cDNA libraries which were incorporated into a set of UniGene clusters that mapped to genome and that represented 41 purely drought responsive and 24% mixed content suggests high sorghum genomic variation related to tissue specific gene expression which are implicated in ecological and evolutionary significances.

Locating protein coding genes using in silico tracing is probably the most difficult, but reliable task of genome annotation and comparison [38]. The need for annotation comparison is not just restricted to different versions of annotation of the same genome but of different sources derived from distinct gene prediction pipelines [39]. Since gene structure prediction is not just a one time complete endeavour that exhaustively describe all possible gene sets in the genome, a long term dynamic process of a variety of efforts requires for progressive update of genome annotation. This endeavour is already subject to change in different organisms via the use of new data repositories and tools publicly available [40]. In this study, the main source of annotation update was an incorporation of additional expression data that signified a modification of one or multiple EGMs. Variability in the genomic features associated with diversification of tissue-specific expression patterns of protein coding genes and the resulting changes in protein function may be a biological implication of such modifications. Merging genes based on multiple overlapping transcripts and novel exonic and UTR features contributed to the improvement of annotation. The identification of novel coding and untranslated part of the existing gene structure models in this study contributed to the dynamic process of sorghum genome annotation. The inclusion of novel structures on a total of 3274 genes with 59 novel exons, 72 putative 5’ UTRs and 3499 3’ UTRs accounted for 9.6% of genome annotation update. The large number of novel identified 3’ UTRs in this study may likely be associated with tissue-specific alternative splicing events and multiple functional polyadenilation [41]. Because, 3’ UTR is the site for regulatory elements including miRNAs and RNA-binding proteins and other stability determining regions, identification of the 3’ UTR is useful to investigate post-transcriptional regulation [42]. It has already been demonstrated that the annotation of 3′ UTR has expanded the scope of post-transcriptional regulatory both in mammals and plants [41, 43].

The finding of drought related 210 putative novel genes with complete, semi-complete and partially truncated but with the start and stop codons and with single exonic feature, contributed to the improvement of the sorghum genome annotation, thus furthering our understanding of sorghum genomics. Identification of the NGSMs with compete structures is an implication of potentially featured new functional elements of the genome annotation, while the truncation may be referred to an in-frame stop codon [44] or often exhibited in the nature of the test dataset.

A recently known prokaryotic characteristics of certain eukaryotic genes is thought to play role in our understanding of the evolutionary patterns of related genes and complex genomes. Such a characteristic feature is evident in the intronless genes in eukaryotic genomes as reported over the past few decades [45]. Furthermore, species-specific intronless enriched genes were shown in Arabidopsis, Oryza, and Populus [26]. A 50% intronless single exonic genes that were shown in our result of which 70.4% were drought responsive was concordant with the already published works in plants for DREB1 intronless expressed gene functionally associated with increased drought tolerance [46]. However, in our analysis, we noted the frequency of intron loss genes to correlate with the processed pseudogene abundance in which case, the latter would be seen as a novel strategy to test the reverse transcriptase model of intron loss [26]. With functional defunct due to frame shifts mutation, interrupted stop codon and gaps within conserved regions, pseudogenes are grouped into processed, duplicated (also unprocessed) and unitary [47]. Even-though further investigation is obligatory, we do however, suspect the presence of pseudogenes from this result in correlation with the finding of single exonic intronless genes in reference to the ENSEMBL consensus criteria for pseudogene [27]. Blastp results revealed 53% of the protein sequences from the novel genes that matched protein domains with known function. However, based on the pfam result, there were still 12% of protein domains annotated as “domains of unknown function” (DUFs), suggesting the novelty of the proteins as well as the importance of experimental research for functional analysis.

Comparative genomics provided opportunities to investigate genome structures and associated features such as alternative splicing, exonic variances and untranslated parts by tracing homology based similarities and differences between organisms [48]. While there is low level of alternatively spliced genes in plants probably for reasons related to plant evolution as compared to animals [49], the identification of 136 alternative splicing in our results suggest the importance of splice event in the regulatory mechanism of gene expression in sorghum crop. As such, alternate exon, in our finding is related to an increase in coding diversity within genes coding for extracellular matrix proteins [50] and in the variability of transcripts. However, it should also be noted that in most cases it may cause unprecedented disorders without the occurrence of splice events [51].

The complete sequencing and annotation of the sorghum genome allows for assigning the coding regions where the majority of genes encode products with known metabolic and biochemical functions [52]. The use of expression data mapping to the sorghum genome allowed identification of metabolic pathways related to drought tolerance and the associated genes for which enriched drought related GO-terms were assigned. In that regard, the identification of glucosinolate biosynthetic pathway among others signify sorghum ability to synthesize and store dhurrin in the tissues and leverage endogenous turnover pathway recycling the nitrogen bound in dhurrin unlike most plants without any effect of the toxic cyanide released into the cell [53]. It was shown that sorghum dhurrin content in leaf tissue is controlled by genes involved the biosynthetic and catabolic pathways in different level of Nitrogen [54]. It was also reported that there is association between high leaf dhurrin content and expression of the stay-green trait [55]. An enzyme CYP79A1 [EC:1.14.13.41] that is grouped into a class of oxidoreductases and encoded by a putatively uncharacterised hypothetical protein gene ‘Sb01g001200’ was identified with a direct involvement in drought tolerance, as it was recently known to be aligned with dhurrin QTL that is associated with stay-green trait [55]. A transcriptional regulation of this enzyme largely determines the synthesis of dhurrin, based on the developmental stage and growth condition of sorghum [53, 56].

A closer analysis of the three pathways namely PCAB, VLIB and VLID shows their integrative metabolic role coordinated by a group of genes that are actively involved in sorghum drought tolerance. Further examination of the biochemical and metabolic pathways shows that these group of genes may seem to be involved in multiple metabolic roles signifying cross-talk between signalling pathways.

Interpro-domain analysis revealed high frequency of protein domains related to drought tolerance such as zing finger domain representing common elements in drought stress response in plants [57] and Chaperon DnaJ doamin protein suggesting functional role in the cooperation of Hsp40 with Hsp70 [58] and in intracellular or endosomal trafficking [59]. Heat shock protein, a ubiquitous molecular chaperon in plants are known to be induced by a wide variety of stresses other than heat shock, including drought [60].

Analysis of gene expression is a vital means of interpreting gained information to discover and develop defensive process in complex trait controlled systems and to disclose polygenic and pleiotropic networks that modulate systems functioning to accurately classify gene features [61]. Moreover, this approach can be used to prioritize a candidate gene list that would otherwise have been a difficult task to assign functionality to genes [61]. In this study, sorghum and maize expression data analysis, revealed a total of 127 prioritized and significantly expressed sorghum genes in association with drought tolerance, concordant with the published work [9, 29].

While the value of orthologous groups is largely noted in illustrating the underlying evolutionary relationship between genes and or protein and in comparative genomic studies, it is also highly recognisable in genome annotation and the identification of candidate genes. The present orthology analysis provided huge over-representation of genes associated with drought tolerance that are prioritized and functionally enriched orthologs.

Integration of genomic information from the current finding with the existing sorghum quantitative traits provided options for identification of the co-localized regions in association with drought tolerance. Detection of the most probable location of QTLs by this method allows determination of the genomic distribution of QTLs known for drought response and the gene-rich-regions [62]**,** providing significant implication on crop improvement. The co-localization of multiple DRGs with several major QTLs controlling drought related and agronomically useful traits provides important information in developing drought tolerance in sorghum which is also useful for understanding the genetic mechanisms underlying this complex trait.

## Conclusions

Detection and functional annotation of the biologically plausible candidate genes in this study required the use of a multi-pronged analytical approach. The reliability and validity of our data contributed to the identification of a large array of functionally enriched DRGs which were not ascribed in previous annotation. The pipeline designated for the identification of DRGs employed multiple informants and standard quality control, which resulted in an update of 9.6% of the existing sorghum genome annotation and an incorporation of 0.6% new information.

Expression profiling and comparative genomic analysis contributed to the identification of orthologous groups that showed high gene conservation along evolutionary lineage with higher shared functional features in ancestrally closer species. The metabolic pathways identified, suggest sorghum’s C4 photosynthetic peculiarity, dhurrin synthesis and other essential characteristics which allow biochemical reactions that make up the metabolic network, constituting a fundamental interface for building sorghum defence mechanism against drought stress.

While this dataset represents a potential source of information that contributes to the field of sorghum genomics which provides insight into enhancing drought tolerance, yet untapped natural genetic variation is certainly evident entailing the need for future research work.

## Methods

### Data acquisition: Reference genome and experimental data

Sorghum genome sequence, UniGene, ESTs and TIGR transcripts and RNA-seq data were used to identify DRGs (Additional file 2: Table S1, Table S13). Genome assembly (sbi1, fasta format) and annotation data (sbi1.4, GFF file) were downloaded from the phytozome database [63] (Additional file 2: Table S1, Table S13) in bulk with 10 chromosomes and 3394 super-scaffolds (small unmapped pieces of genome, that may or may not contain annotated genes and coordinates). The genome is represented with 697,578,683 base pairs arranged in *2n = 20* chromosomes, 34,496 loci containing protein-coding transcripts and 36,338 protein-coding transcripts [8].

A total of 199,087 UniGene sequences (build#30) were retrieved from the National Center for Biotechnology Information (NCBI) UniGene database (Additional file 2: Table S1, S2 and S12) of which 14,057 sequences uniquely represented clusters of UniGenes containing information such as map location and the tissue types where the genes have been expressed [64]. A total of 20,199 drought related ESTs were downloaded from the EST database (dbESTs [65]; Additional file 2: Table S1, Table S13). Based on the information on EST data generated from drought stress experiments under differential expression, 36 libraries were treated with water-stressed conditions at the pre-flowering developmental stage, while 56 were treated under drought stress at the post-flowering developmental stages targeted for stay-green traits. Sequences of a mixture of poly(A) + RNA were organized in a total of 92 normalized cDNA libraries made of 48 body sites and 44 developmental stages of plant tissues grown under differential conditions (Additional file 2: Table S2). A total of 209,835 drought responsive EST transcripts were obtained from the TIGR plant transcript assembly database (the Gene Indices at Dana Faber or the PUTs at Plant GDB) [66] and were cross-checked for redundancies with dbEST from NCBI (Additional file 2: Table S1, Table S13). To detect sorghum DRGs and their orthologs in maize, we retrieved sorghum [9] and maize [29] RNA-seq data generated under drought stress from the GEO (Additional file 2: Table S13).

### Pre-processing (quality filtering process)

Genome and EST sequences were screened for repeats, low complexity and vectors using RepeatMasker v. 3.0 [67]. A run of single pyrimidine or purines were identified using the DUST program [68]. Drought response phenotype information was obtained from the EST library description field to label ESTs within a UniGene cluster as a DREST. For the purpose of this study, UniGene clusters were classified as follows: (i) DREST-only – all ESTs in the cluster were DREST, (ii) non-DREST clusters – none of the ESTs in the cluster were DREST and (iii) a mix of DREST and non-DREST.

### Mapping experimental data to reference genome

We aimed at identifying and characterising known or putatively uncharacterised genes using experimental data obtained from drought responsive libraries. The sorghum genome file was partitioned into its respective chromosomes (1–10) and more than 3300 super scaffolds using an in-house python script. The partitions were used to minimize the size into each chromosome when mapping experimental sequences to the genome. The pipeline presented in Fig. 2 represents a work flow for identifying known and novel CDRGs and annotation updates employing multi-algorithms that include but not limited to BLAST, EXONERATE, AUGUSTUS, BLAT and PASA. The UniGene dataset and the TIGR ESTs were mapped to the sorghum genome in a two step approach: (I) UniGene dataset containing drought ESTs were mapped to the sorghum genome using EXONERATE and BLAT (Fig. 2) whereby coordinates of sequences that mapped to known genes were used to identify DRGs and those to intergenic regions were used as HINTs for AUGUSTUS. (II) UniGene dataset and the TIGR ESTs were mapped to the sorghum genome using BLAT and then valid alignments were assembled by PASA to improve the existing gene annotations (Fig. 2).

Among the 14,057 UniGene clusters used as query sequences, 10,619 were mapped to the reference genome using EXONERATE (Additional file 2: Table S14) and were used in further analysis of genome reannotation. Of these that mapped to the genome at a threshold level of ≥80% identity (Additional file 13 and Additional file 14), 9763 overlapped with the known gene of which UniGene clusters that represent purely DRGs and relatively short DRESTs that were dispersed within the clusters (Additional file 2: Table S3) were identified. All DRGs were functionally annotated (Additional file 2: Figure S1). Existing sorghum gene annotations were functionally characterised as hypothetical, putatively uncharacterised or unknown proteins. The identification of drought responsive transcripts that overlap these existing annotated genes adds drought information and provides additional annotation coordinates that can potentially rectify sorghum gene annotations against EGMs (Fig. 1 and Additional file 2: Table S6).

A total of 209,835 TIGR transcripts DRESTs and 10,619 UniGene clusters (Additional file 2: Table S1) were cleaned by a program called SeqClean and then aligned to the sorghum genome using the PASA pipeline. The main input parameter for SeqClean was a transcript fasta file, but with the vector sequence database, the cleaning process screens for vector by running ‘seqclean transcripts.fasta -v vectors.fasta’. PASA pipeline uses mainly the genome sequence, the 2 SeqClean output files (transcripts.fasta.clean and transcripts.fasta.cln), original and updated annotation files in gff3 format and configuration files for alignment assembly and annotation compare. For instance, the following parameters are typically used for running alignment assembly, “Launch_PASA_pipeline.pl -c alignAssembly.config -C -R -g genome.fasta -t all_transcripts.fasta.clean -T -u all_transcripts.fasta -f FL_accs.txt --ALIGNERS blat,gmap --CPU 2”. The first step of the PASA pipeline uses BLAT, a pre-installed program required by PASA to align transcripts to the genome. A built-in assembly function within PASA was triggered after the transcripts were aligned to the genome and resulted in 5970 assemblies out of 16,835 validated TIGR transcripts DREST alignments and 749 PASA assemblies from 756 validated UniGene cluster alignments. The PASA assembly was undertaken once after clustering the alignments into groups and reassigning them using the validated coordinates of the alignments. Transcripts that aligned to the genome were retained if they met the following threshold: greater than 95% identity and 90% alignment coverage. PASA output includes 520 GFF formatted ICGBs and 500 TRANSDECODER produced BCORFs that were mapped to intergenic region. These transcripts were extracted and analysed according to the procedure outlined in the methods and were used by BLAT to generate the “HINT” files for AUGUSTUS to build gene models and were also involved in annotation modification.

### Building gene models in the intergenic regions

UniGene sequences and TIGR transcripts were aligned against the genome using the following parameters: an e-value cutoff 1e-10; HSPs or hits with at least 80% identity over the entire length of query. HSPs corresponding to the same query were retained if they span a maximum of 2000 bp. Raw blast output was parsed using an in house perl script to identify UniGene sequences that overlapped existing gene annotations and those sequences that mapped to intergenic regions. The latter were retained even if these sequences did not correspond to DRESTs.

**Fig. 9:**
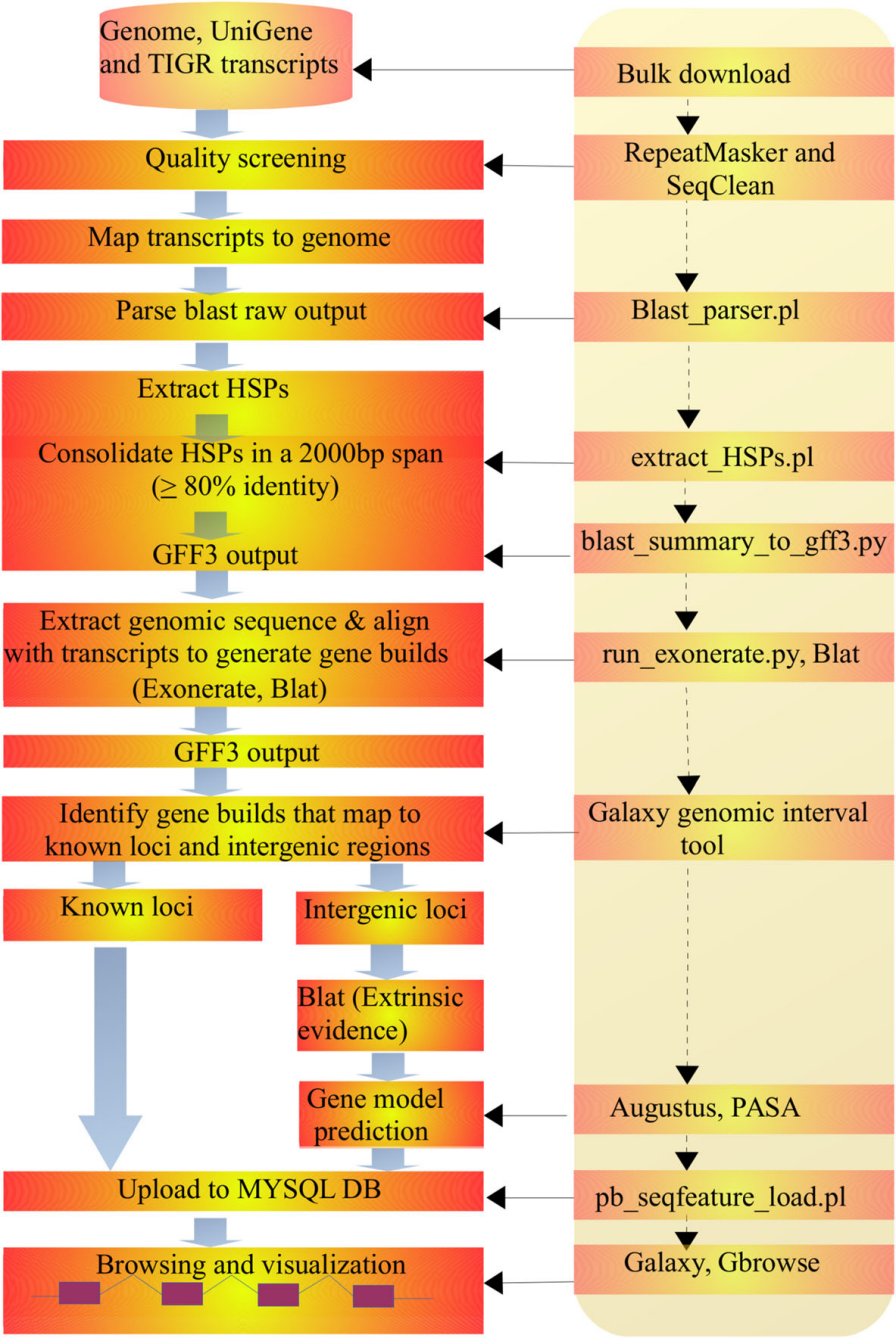
Pipeline for building gene structure models. Drought responsive genes were mapped to sorghum genome using UniGene clusters and TIGR transcripts. Sequences were downloaded as described in the method and were screened for quality using RepeatMasker and SeqClean. These were mapped to genome using e-value cutoff 1e-10. The raw out put was parsed and HSPs were extracted using in-house perl script. Percent identity with ≥80% was used to select the HSPs which were further consolidated along the genomic length of 2000 bp as described in the method. These were converted into GFF3 formats to extract associated genomic region that was aligned to the corresponding transcripts using EXONERATE and Blat to generate gene builds. Known and novel gene builds were classified by intersecting and subtracting the data sets respectively using galaxy genomic interval tool. Gene models were identified by AUGUSTUS and optimized by PASA (Additional file 2: Table S14). Finally, genes were visualized by loading the GFF3 formatted files onto the MySQL DB

The genomic coordinates of these HSPs were extracted and converted to GFF3 format using an in-house perl script. The output was summarized by a python script and was used as an input by EXONERATE, a generic sequence alignment tool that allows rapid implementation of heuristic approximation to exhaustive complex alignment model [22]. The genomic segments from the masked genome were extracted and aligned with the corresponding UniGene sequences by running EXONERATE with these parameters: exonerate --model est2genome query.fasta target.fasta, where the query is the UniGene sequence and the target is the genome. The resulting genomic coordinates were converted to GFF3 formatted file (Fig. 9). These GFF3 formatted UniGene file and the sorghum genome annotation GFF3 file were loaded to the galaxy genomic suite [69] using the “Get Data” option. The UniGene dataset was compared with the genome annotation to find the known genes that correspond to the mapped UniGene sequences using the “Compare two Datasets” option. Intergenic (novel) loci were identified using the “Subtract Whole Dataset” in the galaxy genomic suite.

The GFF3 formatted output of 856 BCUCs of which 128 were purely drought responsive that were filtered from a total of 1067 UniGene clusters that mapped to intergenic region were used as additional input to BLAT to generate HINT. This was then used by AUGUSTUS along with the sorghum genome and expression data, sorghum parameter and meta parameter following an established procedure in [21] as extrinsic evidence in gene prediction.

### Annotation comparison and updates

One of the modifications in the existing annotation was the change in structural and positional categories of the EGMs, which we described as follows: I) *Bidirectionally extended overlapping genes*: A set of predicted genes that overlapped with the EGMs having 3′ and 5′ ends extended in both direction over the EGMs; ii) *Unidirectionally extended overlapping genes*: genes that overlapped with the EGMs and unidirectionally extended just on one of either ends (3′ or 5′) but not both; iii) *perfect overlapping genes*: genes that exactly match the coordinates of the EGMs; iv) *partial overlapping genes at the 5′ end*: genes that shared the start coordinate with the EGMs. v) *partial overlapping genes at the 3′ end:* genes that shared the 3′ end with EGMs; vi) *Inner overlapping genes:* genes that fall exclusively within the range of the EGMs (Fig. 1a). vii) *cross-genic overlapping (merging) genes*: genes that overlapped or shared with the coordinates of more than one EGMs (Fig. 1b); viii) *non-overlapping (novel) genes*: genes that fall exclusively outside of the range of the EGMs mapping to intergenic regions (Fig. 1c).

The PASA pipeline was used to compare the existing sorghum genome annotation with the new genome mapping coordinates derived from the DRESTs. This is because PASA, of the available tools, can be used to report differences between existing and newly created annotations [70]. Table 4 shows the parameters set in the PASA pipeline for the annotation comparison and minimum full length ORF size. Based on these parameters, all the valid single gene model updates that retained PASA assembly reference id were computed and compared with the non-modified original gene structure. We used the term “update” to explain annotation modification that depicts addition of new features resulting in structural improvements by extending genomic coordinates on anyone or multiple genes in the form of complete or partial transcripts, exons, CDS and UTRs of the EGMs based on expression data used.

**Table 4:** Summary of parameters used in the PASA pipeline for the annotation comparison and minimum full length ORF size

Annotation comparison		Minimum full length ORF size	
Parameters	Minimum %	Parameters	Minimum value
Genomic overlap	50	Annotation version	2
Protein coding	40	Maximum utr exons	2
Length for non-full-length compare	70	Compare ID	2
Length for full-length compare	70	Trust full length status	0
Predicted protein compare	70	stomp	0
Alignment length	70	Minimum % overlap	80

The annotation update in this study includes: (1) modification of the existing annotation, and (2) discovery of novel loci. The PASA pipeline uses built-in dependency alignment tools such as BLAT, GMAP and BLAT-GMAP as default aligners, however, in this prediction, BLAT was used because of the reasons outlined below. The default values used for the thread number of the pipeline, the number of top scoring spliced alignments and the minimum % overlap of the transcripts to be clustered were equivalent to ‘2’, ‘1’ and '30' receptively.

### Prediction of gene structure models using AUGUSTUS

BLAT was used as a dependency alignment tool both for AUGUSTUS and PASA, because of it’s greater accuracy and faster speed than existing tools. It uses ‘-ooc = 11.ooc’ option that tells the program to load over-occurring 11-mers from external file which basically increases the speed by a factor of 40 [23]. For mRNA/DNA alignments, BLAT allows extension of all perfect hits, stitches homologs into single larger alignment unsplicing mRNA on to the genome that uses each base of the mRNA only once which correctly positions splice sites [23]. Based on this, the three types of initial gene set were used by BLAT to generate HINTs. BLAT initially produced “*.psl” formatted file of a DNA sequences homology with ≥95% identity and the default coverage of 80%. This was sorted by using pslSort program and command line “sort -K 10,10”. The sorted output was used by pslReps to select the best alignments which were finally subjected to pslCDnaFilter, a standard tool of the University of California, Santa Cruz (UCSC), to filter again the alignments and report only the top HSPs for each input sequence before the last run of BLAT to create HINTs. The setting of parameters for pslCDnaFilter was based on the EST/mRNA of the UniGene track construction protocol given in BLAT software (− minId = 0.95 – minCover = 0.25 – localNearBest = 0.001 – minQSize = 20 – minNonRepSize = 16 – ignoreNs – bestOverlap – polyASizes = ployAFile, where polyAFile was generated by UCSC program faPolyASizes). The HINTs, were then produced by BLAT using AUGUSTUS utility, script blat2hints.pl.

AUGUSTUS, a stand-alone software, uses the following parameters for running gene prediction: AUGUSTUS –species = species –hintsfile = hints.E.gff –extrinsicCfgFile = extrinsic.ME.cfg genome.fa. Species and genome were set to represent sorghum according to the options given in the program. HINTs were separately used by AUGUSTUS as experimental evidence to predict the gene structure models, then the outputs were pooled together. AUGUSTUS either accept or ignore a HINT depending on the level of its compatibility and reliability to predict gene structure [24] whereby predicted genes were assigned to ab initio for HINTs which were not compatible. A combination of ab initio and homology based prediction were used to identify potential novel candidate genes.

#### Consistency in gene predictions

The consistency in gene prediction was checked using multiple data sources selected based on sequences mapped to the intergenic regions. The results in the bitscore in AUGUSTUS prediction from each datasets were compared and the evidence support were used to show consistency in gene prediction. These were used to evaluate the novelty of gene structure models in combination with the parameters used for screening gene models.

### Filtering the gene structure models

The following parameters were used to filter the NGSMs: i) genomic coordinates of the novel genes in relation to the intergenic distance between nearest neighbouring EGMs or the predicted genes if they were neighbours; ii) length of the predicted genes; ii) score of the predicted genes; iv) percentage evidence support where prediction was homology; v) Strand orientation of the predicted genes in relation to the existing genes or the currently predicted genes if they were in close proximate. The parameters are not necessarily in order of their weight, however each of these contributes to the novelty of the gene structure models. We cautiously used the genomic coordinates as the primary and mandatory screening parameter to make sure none of the novel genes has an overlapping coordinate with the EGMs. Coordinates for all known sorghum genes were obtained from phytozome (release v3.1, v2.1, Sbi1.4) to compare with the genomic coordinates of the AUGUSTUS gene models. This was done only after the NGSMs were optimized by PASA because the optimization step updates the gene models and may lead to the change in the genomic coordinates. Genes satisfied any of the four listed criteria were considered valid leaving genomic coordinates and length of the gene models as mandatory. Manual curation and post PASA update functional annotation of the NGSMs were conducted.

### Optimization of gene prediction

The best scoring candidate gene models, AUGUSTUS GFF3 format, were modified by PASA pipeline utility code to meet compatibility with PASA pipeline environment. These were then subjected to PASA reprocessing step to generate updated final set of gene models which were further evaluated for optimal structure model with UTRs and ASVs prediction and fitting all best model to the splice sites.

### Functional annotation of genes identified

Drought responsive novel gene structure models were filtered and subjected to post-gene-prediction process to functionally annotate. Non-redundant protein database search was conducted using BlastP (protein-protein blast; [37]) to determine the type of proteins to which they best mapped (Additional file 4). We used 1e-10 as an e-value cut-off for the protein-protein blast. The best blast hits were filtered based on the bit score value. Protein query sequences that mapped to known proteins were identified and those which were not mapped to any know protein database but remained unique to sorghum was also identified. In addition, we conducted analysis of pfam to identify the conserved protein domains and associated annotation based on the protein sequences identified for drought responses using default parameters (Additional file 5).

### Metabolic pathway analysis

Biochemical pathway analysis was performed using the KEGG database [71] which is supported by BLAST2GO database and software [72]. A total of 123 UniGene sequences that mapped to the sorghum genome and overlapped with known genes were searched against the BLAST2GO databases using the BLASTX [37] search algorithm using default e-value cut-off parameter (1e-10). The number of hits and the HSPs length cut off value per query sequence were set to 50 each. Enzyme Code (EC) weight was set to 1 or 0 depending on whether the influence of the evidence codes on the GO annotations is required or ignored (eg. IEAs) respectively. A list of EC, KEGG pathway maps, interpro annotation and statistics, GO annotation and combined graphs for GO-categories of Biological Process (BP), Cellular Component (CC) and Molecular Function (MF) were identified. Sequence distributions based on blast hits associated with the GO-terms for the biological process is shown in Additional file 2: Figure S4a-d. Gene enrichment analysis for genes mapped to metabolic pathways and Interpro-domains was carried out as described in the BLAST2GO based GO enrichment protocol.

### GO functional enrichment analysis using BLAST2GO

GO functional enrichment and annotation for the UniGene sequences that overlapped with the known genes was performed using BLAST2GO and was configured to e-value cut-off <1.0e-6. Default values were used for the annotation cut-off = 55, a GO-weight = 5. We used HSP-hit coverage = zero, because, HSP-hit coverage greater than zero may create chances of missing any best hit from the HSP spans [72]. Once setting the parameters, BLAST2GO employed a BlastX program, to search for matching nucleotides against NCBI non-redundant database. Each UniGene/EST sequence was assigned with a GO term and an Interpro-domain identifiers. The occurrence of GO terms assigned to each UniGene was compared to the one of the background set of GO-annotated transcripts in the entire database using the hypergeometric distribution. Gene ontology domains namely BP, CC and MF based tree-type combined-graphs were configured using default values provided by BLAST2GO for all enriched GO terms (adjusted *p*-value <0.05). Mapping was performed to associate the blast HSP-hits to functionally enriched information from GO DB. All annotations are associated to an evidence code which provides information about the quality of this functional assignment. Default parameters were used to assign Interpro-domain and GO terms to the identified gene models. Sorghum peptides were selected for the occurrence of functional motifs and protein signature for which statistical significance of over-representations of each GO term exist. Enrichment status of the GO terms were checked using Fisher’s exact test in comparison to the background set based on *p*-values. The gene set with lowest p-value represent the significance level of enrichment. Terms representing all the GO categories were used in annotation for the enriched ones with adjusted *p*-value (FDR, *p* < 0.05).

### GO functional enrichment analysis using AGRIGO

GO enrichment analysis for candidate known genes identified by BLAST sequence similarity search based on mapping UniGene clusters to sorghum genome (Additional file 15) was performed using AGRIGO [73], a web-based tool and database for the gene ontology analysis. This was compared with the result performed using BLAST2GO. Query sequences of a total 123 known genes that matched the same total (123 UniGene clusters) were used as an input for AGRIGO to evaluate the genes to which the enriched GO terms were assigned (Additional file 15). These genes then compared to the total number of genes obtained the Interpro information from the BLAST2GO analysis.

Similarly, GO enrichment analysis for the genes identified by the other two underlying approaches (analysis of expression profiling and orthologous groups) were performed using AGRIGO separately after the candidate genes were identified by each approach. Singular Enrichment Analysis (SEA), a version of Gene Set Enrichment Analysis (GSEA) [74] was performed based on enrichment of the GO terms obtained after comparing the statistical test with pre-calculated background set. GO term enrichment and the number of genes mapped to the enriched terms were determined by Parametric Analysis of Gene Set Enrichment (PAGE) using a Z-score value which eventually was converted to the p-value for correction inferring the statistical significance of the GO term enrichment. AGRIGO allows checking for enrichment status of GO terms using Fisher’s exact test as a default against the background set based on *p*-values. Adjusted p-value, FDR, *p* < 0.05 was used to determine the significance level of enrichment. The gene set returned with p-value lower that 0.05 were retained.

The final set of genes associated with all GO-terms with direct or indirect correlation with drought stress responses were selected based on the BP, CC and MF. The GO term descriptors that related to drought tolerance were used to select the CDRGs (Additional file 2: Figure S17). Mapping of the GO-terms related to responses to stress based on biological processes was configured by tree traversing mode.

### Gene expression profiling

To investigate potential candidate genes that respond to drought stress conditions in sorghum, we analysed the gene expression data generated under drought stress from sorghum [9] and maize [29] separately. Sorghum and maize RNA-seq expression data associated with drought stress was retrieved from NCBI, GEO database [75] to identify tissue-specific pattern of gene expression (Additional file 2: Table S13). Based on the list of maize genes generated under drought condition for fertilized ovary and leaf meristem tissues, we identified sorghum orthologs using ortholog pairs recorded in the ENSEMBL Biomart database [31]. The raw expression data from both species was analysed separately using parametric t-test (*P*-value <0.01) for sorghum genes and three independent statistical methods for which the significance was compared for sorghum orthologs to see if additional drought responsive genes were identified.

A software package, TIGR Multiple Experiment Viewer (MeV; MeV4.8.1) [76], was used to analyse the differentially expressed genes. Sorghum and maize genes that were over-expressed (≥2-fold RNA-seq) under drought stress were visualized separately as the heat maps (Figs. 5 and 6). Volcano plots were used to show the up and down-regulated genes based on expression threshold level (Additional file 2: Figure S13; Figure S14). The over-expressed genes corresponding to the two species were used as an input separately into AGRIGO [73] to determine their functional correlation with drought responses based on GO term enrichment (FDR, *p* < 0.05).

### Statistical analysis of gene expression

Multivariate analysis of variance was used to identify statistically significant over-expression of genes under stringent criteria using parametric and non-parametric tests. Significant differences in gene expression levels was evaluated by employing unpaired t-Test to estimate between subject variance. Non-parametric Fisher’s exact test [77] was used to evaluate the effect of treatments on the gene expression outcome, and a FDR calculation [78] for genes identified at *p* < 0.05 were performed. Rank products, a non-parametric statistical method [79] was employed to minimize the discrepancy between the actual and false discovery of differentially expressed genes. Tissue and treatment based groupings of the samples were employed to determine the effect of these parameters on the gene expression. The treatments used in this analysis represent drought stress and well-watered condition while tissue types were root and shoot for sorghum data (Additional file 7) and fertilized ovary and basal leaf meristem for maize (Additional file 8).

### Analysis of orthologous groups

A total of 9693 sorghum UniGene clusters out of a total of 14,057 that contain one or more drought responsive ESTs (Additional file 2: Table S1) was used for orthology analysis. Sorghum drought responsive orthologs were identified in three species namely arabidopsis, rice and maize (Additional file 10) and were retrieved from the ENSEMBL Compara database using ENSEMBL BioMart [31].

Percent identity and orthology confidence levels were used as parameters to retrieve matching orthologs. All available homology types (one2one, one2many and many2many) that have more than 50% identity and high level orthology confidence as a threshold value cut off were considered for selecting the best quality orthologs (Additional file 10). These were used to undertake the GO enrichment analysis using AGRIGO based GO annotation protocol.

### Association of target DRGs with QTLs

In order to identify target DRGs that were associated with different QTLs, we first obtained the genomic location of the QTLs based on the previous studies [33, 80, 81] and compared with the genomic position of the genes currently identified. If the gene coordinates overlap with or fall in the QTLs regions, then we considered that there was high chance that the genes were associated with the QTLs as they were co-localized. Secondly, we extracted the genomic sequences of the QTLs, where the QTLs regions were relatively smaller [32, 34] and aligned with the sequences of the target genes using the program BLASTN [37], to identify the best blast hit based on e-value 1e-10 and % identity >80.

## Additional files

Additional file 1: Description of functional annotation of novel gene structure models. (XLSX 132 kb)

Additional file 2: Table S1. Summary of sorghum transcript and genomic data. **Table S2.** Overview of UniGene libraries (build # 30). **Table S3.** Chromosomal distribution of UniGene clusters mapped to genome. **Table S4.** Comparison and update of annotation. **Table S5.** Functional distribution of the novel gene structure models. **Table S6.** Chromosomal distribution of existing genes modified corresponding to the position and structure of modification. **Table S7.** Chromosomal distribution of the novel gene structure models. **Table S8.** Genomic distribution of spliced and retained intron based on PASA analysis. **Table S9.** Genomic distribution of skipped and retained exons based on PASA analysis. **Table S10.** PASA based identification of alternative splicing (AS) for the novel genes. **Table S11.** Patterns of exonic and intronic features in the novel gene structure models. **Table S12.** GO functional enrichment of DR sorghum genes based on orthology groups. **Table S13.** Databases containing potential candidate DRGs. **Table S14.** Relevant tools for identification of the candidate gene. **Table S15.** Functional description of sorghum drought related metabolic pathways. **Figure S1.** Genes identified by sequence mapping to existing gene models and their annotation status. **Figure S2.** Pattern of exon and intron number and the average length. **Figure S3.** Nearest intergenic distances. **Figure S4.** GO annotation based on blast and mapping to non-redundant databases. **Figure S5.** Aminoacyl-tRNA biosynthesis (upper) and Cysteine and methionine metabolism (lower). **Figure S6.** Drug metabolism - other enzymes (upper) and Glucosinolate biosynthesis (lower). **Figure S7.** Glycerophospholipid metabolism (upper) and Glycerolipid metabolism (lower). **Figure S8.** Phosphatidylinositol signalling system (upper) and nicotinate and nicotinate metabolism (lower). **Figure S9.** Pyrimidine metabolism (upper) and purine metabolism (lower). **Figure S10.** Valine, Luecine and Isoleucine Biosynthesis (upper) and Valine, Luecine and Isoleucine degradation (lower). **Figure S11.** Pantothenate and CoA biosynthesis. **Figure S12.** Description of interpro-domain analysis: List of protein signatures identified. **Figure S13.** Volcano plots for expression profiles based on sorghum drought responsive genes. **Figure S14.** Volcano plot showing differential expression of genes based on maize expression data. **Figure S15.** Venn diagram showing distribution of significantly expressed genes. **Figure S16.** Sorghum % GO-terms assigned to genes identified from maize orthologs. **Figure S17.** Mapping of GO terms related to responses to stress based on biological process. (PDF 1372 kb)

Additional file 3: Description of the novel structure such as mRNAs, exons and UTRs that modified existing gene models. (XLSX 432 kb)

Additional file 4: Description of functional annotation of novel drought responsive proteins using BlastP. (XLSX 47 kb)

Additional file 5: Description of functional annotation of novel drought responsive proteins using Pfam. (XLSX 19 kb)

Additional file 6: Description of metabolic pathways. (XLSX 1641 kb)

Additional file 7: Description of the gene expression profiles for the tissue and treatment related analysis based on sorghum RNA-seq data generated under drought stress. (XLSX 58 kb)

Additional file 8 Description of the sorghum drought responsive orthologs identified based on maize expression profiling from RNAseq data generated under drought stress. (XLSX 31 kb)

Additional file 9: Combined drought related GO terms for Sorghum genes based on sorghum expression data and sorghum orthologs in maize based on gene expression profiles. (XLSX 44 kb)

Additional file 10: Analysis of orthology relationship between sorghum, maize, rice and Arabidopsis based on ensemble BioMart. (XLSX 226 kb)

Additional file 11: Description of drought responsive genes associated with different types of sorghum QTLs based on comparison of the genomic locations. (XLSX 445 kb)

Additional file 12: Description of drought responsive genes associated with different types of sorghum QTLs based on sequence alignment. (XLSX 13 kb)

Additional file 13: Blast result summary. (XLSX 318 kb)

Additional file 14: Description of UniGene to genome mapping. (XLSX 331 kb)

Additional file 15: Functional annotation of DRGs identified by UniGene to genome Mapping. (XLSX 32 kb)

## Abbreviations

BCORFs: Best Candidate Open Reading Frames
BCUCs: Best Candidate UniGene Clusters
BP: Biological Process
CC: Cellular Components
CDRG: Candidate Drought Responsive Genes
DRESTs: Drought Responsive ESTs
DRG: Drought Responsive Gene
EGMs: Existing gene models
ESTs: Expressed Sequence Tags
FDR: False Discovery Rate
GEO: Gene Expression Omnibus
HSPs: High-scoring alignment pairs
ICGBs: Initial Comprehensive Gene Builds
MF: Molecular Functions
NGSMs: Novel gene structure models
PASA: Program to Assemble Spliced Alignment
PCAB: Pantothenate and CoA biosynthesis
TIGR: The Institute for Genomic Research
VLIB: Valine, Leucine, and Isoleucine biosynthesis
VLID: Valine, Leucine, and Isoleucine degradation
