# Supplementary material for: An integrated and comparative approach towards identification, characterization and functional annotation of candidate genes for drought tolerance in sorghum (Sorghum bicolor (L.) Moench)
Source: BMC Genet. 2017 Dec 22;18:119. doi: 10.1186/s12863-017-0584-5 (PMC5741957; doi:10.1186/s12863-017-0584-5)
Supplement: Supplementary file 2 — Summary of sorghum transcript and genomic data. Table S2. Overview of UniGene libraries (build # 30). Table S3. Chromosomal distribution of UniGene clusters mapped to genome. Table S4. Comparison and update of annotation. Table S5. Functional distribution of the novel gene structure models. Table S6. Chromosomal distribution of existing genes modified corresponding to the position and structure of modification. Table S7. Chromosomal distribution of the novel gene structure models. Table S8. Genomic distribution of spliced and retained intron based on PASA analysis. Table S9. Genomic distribution of skipped and retained exons based on PASA analysis. Table S10. PASA based identification of alternative splicing (AS) for the novel genes. Table S11. Patterns of exonic and intronic features in the novel gene structure models. Table S12. GO functional enrichment of DR sorghum genes based on orthology groups. Table S13. Databases containing potential candidate DRGs. Table S14. Relevant tools for identification of the candidate gene. Table S15. Functional description of sorghum drought related metabolic pathways. Figure S1. Genes identified by sequence mapping to existing gene models and their annotation status. Figure S2. Pattern of exon and intron number and the average length. Figure S3. Nearest intergenic distances. Figure S4. GO annotation based on blast and mapping to non-redundant databases. Figure S5. Aminoacyl-tRNA biosynthesis (upper) and Cysteine and methionine metabolism (lower). Figure S6. Drug metabolism - other enzymes (upper) and Glucosinolate biosynthesis (lower). Figure S7. Glycerophospholipid metabolism (upper) and Glycerolipid metabolism (lower). Figure S8. Phosphatidylinositol signalling system (upper) and nicotinate and nicotinate metabolism (lower). Figure S9. Pyrimidine metabolism (upper) and purine metabolism (lower). Figure S10. Valine, Luecine and Isoleucine Biosynthesis (upper) and Valine, Luecine and Isoleucine degradation (lower). Figure S11. Pa [file 12863_2017_584_MOESM2_ESM.pdf]

Additional file 2

Table S1 Summary of sorghum transcript and genomic data

Sequence origin	Sequence type	Number of sequences	GC%	URL
Genome	Chromosomes	10	41.6	http://www.phytozome.net/sorghum
	Super scaffold	3394	40.9	http://www.phytozome.net/sorghum
UniGene	Unclustered UniGene	199087	52.9	http://www.ncbi.nlm.nih.gov/UniGene/
	Clustered UniGene	14057	52.0	http://www.ncbi.nlm.nih.gov/UniGene/
ESTs	TIGR transcripts	209835	53.3	http://www.ncbi.nlm.nih.gov/dbEST/ http://www.plantgdb.org/prj/ESTCluster/ ftp://occams.dfci.harvard.edu/pub/bio/tgi/data/
	NCBI dbEST	20199	50.7	http://www.ncbi.nlm.nih.gov/dbEST/
RNA-seq	Maize GEO data (GSE40070)	3000000 reads	NA	http://www.ncbi.nlm.nih.gov/geo/

Table S2 Overview of UniGene libraries (build # 30)

Main parts of plants	Tissues	Number of lib[a]	Library ID	Name of Library	Sequence number
Body site	Callus	1	Lib.13735	Callus culture/cell suspension	10449
	embryo	2	Lib.5437	Embryo 1 (EM1)	9843
	leaf	12	Lib.15546<br>Lib.10086<br>Lib.14497<br>Lib.14500<br>8 not shown	Wounded leaves<br>Pathogen-infected<br>compatible 1 (PIC1)<br>Drought-stressed after<br>flowering<br>Drought-stressed before<br>flowering	11221<br>9092<br>6295<br>3597<br>each <1000
	ovary	3	Lib.5439<br>Lib.7266<br>1 not shown	Ovary 1 (OV1)<br>Ovary 2 (OV2)	5052<br>4983<br><1000
	panicle	2	Lib.9519<br>1 not shown	Immature panic le 1 (IP1)	9588<br><1000
	pollen	1	Lib.14372	Pollen	10212
	chloroplast	1	not shown		each <1000
	root	3	Lib.15544<br>Lib.16897<br>Lib.20762	Acid- and alkaline-treated<br>roots<br>Anaerobic roots<br>Sorghum bicolor BTx623<br>Root hair	7744<br>6113<br>5468
	shoot	2	Lib.16898<br>1 not shown	GA- or brassinolide-<br>treated seedlings	11134<br><1000
	Whole	4	not shown		each <1000
	Unspecified tissue	1	not shown		<1000
	mixed	13	Lib.4037<br>Lib.13713<br>Lib.15545<br>Lib.4038<br>Lib.13770<br>Lib.13736<br>Lib.13769<br>Lib.12996<br>Lib.14109<br>Lib.14297<br>Lib.14296	Dark Grown 1 (DG1)<br>Heat-shocked seedlings<br>Oxidatively-stressed<br>leaves and roots<br>Water-stressed 1 (WS1)<br>Ethylene-treated seedlings<br>Salt-stressed seedlings<br>Salicylic acid-treated<br>seedlings<br>Abscisic acid-treated<br>seedlings	11099<br>10558<br>10086<br>10039<br>6942<br>6737<br>5801<br>4907<br>3984<br>3849<br>3723

**Main parts of plants**	**Tissues**	**Number of lib**[a]	**Library ID**	**Name of Library**	**Sequence number**
			2 not shown	Iron-deficient seedlings<br>Nitrogen-deficient seedlings<br>Phosphorous-deficient seedlings	each < 1000
	not yet classified	4	Lib.5441<br>Lib.2801<br>2 not shown	Pathogen induced 1 (PI1)<br>Light Grown 1 (LG1)	9569<br>9451<br>each <1000
Developmental stage	Germinating	2	Not shown		each <1000
	seed	25	Not shown		each < 1000
	seedling	9	Not shown		each < 1000
	vegetative	1	Not shown		< 1000
	flowering	1	Not shown		< 1000
	ripening	1	Not shown		< 1000
	unknown	3	Not shown		each < 1000
	developmental stage not yet classified	2	Not shown		each < 1000

Table S3 Chromosomal distribution of UniGene clusters mapped to genome

Chromosomes	UniGene clusters that overlapped Known genes			UniGene clusters that mapped to intergenic region			Grand Total
	DR[1]	Non-DR(DR)[2]	Total	DR	Non-DR	Total	
Chr1	20	1733 (37)	1753	22	91	113	1866
Chr2	14	1181 (33)	1195	20	69	89	1284
Chr3	16	1322 (39)	1338	15	73	88	1427
Chr4	13	1150 (35)	1163	12	64	76	1239
Chr5	4	432 (13)	436	7	49	56	493
Chr6	19	876 (21)	895	10	64	74	966
Chr7	9	670 (23)	679	9	61	70	749
Chr8	8	544 (13)	552	7	63	70	622
Chr9	14	883 (27)	897	12	66	78	976
Chr10	6	750 (15)	756	7	54	61	817
Super	0	99 (2)	99	7	74	81	180
Total	123	9640 (258)	9763	128	728	856	10619

Key to legend:

[1] UniGene clusters that represent purely drought responsive

[2] UniGene clusters that basically represent non-drought responsive, however, contain shorter ESTs which were expressed under drought conditions dispersed within the clusters.

Table S4 Comparison and update of annotation

Description	Existing annotation		Current annotation			
			Updated		Novel	
	gene models	mRNA	gene models	mRNA	gene models	mRNA
Original	34211	37205	30937	33862	-	-
Updated	-	-	3274	3343	210	210
Total	34211	37205	34211	37205	210	210

This supplementary table shows the comparison and update of annotation between existing and current prediction of sorghum genome based on PASA alignment evidence.

Table S5 Functional distribution of the novel gene structure models

Known drought-related function	Complete genes		Partial genes			Total
	3' and 5' UTR attained	only 3' UTR attained	only 5' UTR attained	at-least one end attained	truncated at both ends	
DR	12	15	2	29	137	166
NDR	0	0	0	0	44	44
Total	12	15	2	29	181	210

Table S6 Chromosomal distribution of existing genes modified corresponding to the position and structure of modification.

Scaffolds	Chromosomal distribution of the modified existing gene models									Total
	A[1]	B[2]	C[3]	D[4]	E[5]	F[6]	G[7]	H[8]	I[9]	
chr1	3	16	24	642	0	0	1	0	0	685
chr2	6	11	7	373	0	0	0	1	0	397
chr3	2	19	14	445	0	0	0	1	0	480
chr4	2	13	11	402	0	0	1	0	0	428
chr5	2	3	7	170	0	0	0	0	0	182
chr6	2	8	5	220	0	0	0	0	0	235
chr7	0	5	10	193	0	0	0	0	0	208
chr8	0	4	4	182	0	0	0	0	0	190
chr9	3	2	3	254	0	1	0	0	0	263
chr10	1	4	11	248	0	0	0	0	1	265
super	0	0	0	10	0	0	0	0	0	10
Total	21	85	96	3139	0	1	2	2	1	3343

Legend to key:

A[1] Transcript A; B[2] Transcript B; C[3] Transcript C; D[4] Transcript D; E[5] Transcript E; F[6] Transcript F; G[7] Transcript G; H[8] Transcript H and I[9] Transcript I (Figure 1)

Table S7 Chromosomal distribution of the novel gene structure models

Scaffolds	Complete gene structure		Partial gene structure				Total
			One end retained		truncated at both ends		
	DR[a]	ND-DR[b]	DR[a]	ND-DR[b]	DR[a]	Non-DR[b]	
chr1	3	0	0	0	12	13	28
chr2	3	0	0	0	9	8	20
chr3	1	0	2	0	15	3	21
chr4	1	0	4	0	10	8	23
chr5	0	0	2	0	11	6	19
chr6	0	0	1	0	8	3	12
chr7	1	0	4	0	10	5	20
chr8	2	0	0	0	16	7	25
chr9	0	0	2	0	16	2	20
chr10	1	0	2	0	11	6	20
super	0	0	0	0	1	1	2
Total	12	0	17	0	119	62	210

Key to legend: [a] Drought responsive; [b] Non-drought responsive

Table S8 Genomic distribution of spliced and retained intron based on PASA analysis

S[1]	Intron							
	Spliced				Retained			
	Coordinate	O[2]	A[3]	M[4]	Coordinate	O[2]	A[3]	M[4]
chr1	1819870-1820018	+	64	1	1819870-1820018	+	65	1
chr1	2245082-2245182	-	83	5	2245082-2245182		84	2
chr1	2630315-2630410	-	101	1	2630315-2630410	-	102	2
chr1	8195826-8195903	-	306	1	8195826-8195903	-	307	1
chr1	47862385-4786251	+	638	6	47862385-4786251	+	639	6
chr1	56966890-56966985	-	779	2	56966890-56966985	-	780	1
chr1	58887490-58887568	-	819	3	58887490-58887568	-	820	1
chr1	58887601-58887781	-	819	1	58887601-58887781	-	820	1
chr1	61110177-61110310	-	896	9	61110177-61110310	-	897	2
chr1	67980410-67980506	+	1125	6	67980410-67980506	+	1126	2
chr2	69162027-69162097	-	2358	2	69162027-69162097	-	2357	2
chr3	62293574-62293685	-	3153	3	62293574-62293685	-	3154	1
chr4	65061928-65062017	+	4139	2	65061928-65062017	+	4140	1
chr6	45323794-45323870	-	4626	18	45323794-45323870	-	4627	1
chr6	60237462-60237612	+	4942	2	60237462-60237612	+	4943	1
chr7	8345323-8345537	+	5138	5	8345323-8345537	+	5139	4
chr8	6642051-6642134	+	5536	7	6642051-6642134	+	5537	1
chr8	6641856-6641938	+	5536	4	6641856-6641938	+	5537	1
chr8	44687799-44687915	-	5622	1	44687799-44687915	-	5621	1
chr9	2436568-2436638	-	5784	4	2436568-2436638	-	5785	1
chr9	52085543-52085653	-	6061	2	52085543-52085653	-	6060	4
chr9	46038980-46039077	+	5954	32	46038980-46039077	+	5955	15
chr9	46038375-46038506	+	5953, 5954	178	46038375-46038506	+	5955	4
chr9	54606686-54606830	+	6117	2	54606686-54606830	+	6118	2
chr9	57490227-57490310	-	6208	5	57490227-57490310	-	6209	1
chr10	58496008-58496090	-	1717	2	58496008-58496090	-	1718	1

Key to legend: [1] Chromosomes number 1-10; [2] Orientation of the strand; [3] Assembly number that the transcripts belong to and [4] Maximum evidence support.

Table S9 Genomic distribution of skipped and retained exons based on PASA analysis

S[1]	Exon							
	Skipped				Retained			
	Coordinate	O[2]	A[3]	M[4]	Coordinate	O[2]	A[3]	M[4]
chr2	15949877-15955756	+	1983	1	15951004-15951091, 15951181-15951253	+	1984	1
chr2	75790551-75791220	+	2540	1	75790984-75791097	+	2539	7
chr3	71528810-71529369	-	3403	2	71529086-71529156	-	3404	1
chr4	4717042-4717529	+	3609	1	4717411-4717488	+	3608	11
chr6	1517417-1519399	-	4521	1	1518211-1518383	-	4520	2
chr6	60298761-60299726	-	4947	1	60299069-60299251	-	4946	9
chr9	54972512-54974544	-	6127	2	54972660-54973133	-	6126	10
chr10	2956478-2957725	-	1340	1	2956662-2956707	-	1339	1
chr10	59988260-59988901	+	1779	3	59988341-59988410	+	1780	2

Key to legend: refer to Table S6.

Table S10 PASA based identification of alternative splicing (AS) for the novel genes

Types of AS	AS event	Non-AS events
Alternative acceptor	42	-
Alternative donor	14	-
Alternative 5' site	-	-
Alternative 3' site	-	-
Alternative exon	16	-
Ends in intron	10	-
Retained exon	-	9
Retained Intron	27	-
Skipped exon	9	-
Spliced intron	-	27
Starts in intron	18	-
Total	136	36

Table S11 Patterns of exonic and intronic features in the novel gene structure models

Features[a]	Feature less	Single	Double	Triple	Quadruple	Multiple features					Total
						5	6	7	8	9	
Total Exons	0	210	105	62	30	17	8	5	2	2	441
Total introns	0	105	62	30	17	8	5	2	2	0	231
Gene per exonic[b]	0	105[d]	43	32	13	9	3	3	0	2	210
Gene per intronic[c]	105[e]	43	32	13	9	3	3	0	2	0	210

Key to legend: [a] Genes, Exons and Introns; [b] Gene number per exonic feature; [c] Gene number per intronic feature; [d] Single exonic genes and [e] Intronless genes.

Table S12 GO functional enrichment of DR sorghum genes based on orthology groups

GO-D[A]	GO ID	GO-term	E/T[B]	GO/Bg[C]	P[D]
BP	GO:0006950	Response to stress	1102/1102	3705/26245	0.00
BP	GO:0006950	Response to water deprivation	118/1102	374/26245	4.98e-55
BP	GO:0009269	Response to dessication	21/1102	71/26245	5.4e-09
BP	GO:0009408	Response to heat	91/1102	324/26245	1.35e-38
BP	GO:0009737	Response to ABA stimulus	109/1102	664/26245	2.87e-28
BP	GO:0009738	ABA mediated signalling pathway	37/1102	192/26245	4.87e-11
CC	GO:0044444	Cytoplasmic part	477/1102	7660/26245	2.07e-20
CC	GO:0009536	Plastid	161/1102	2109/26245	5.95e-11
CC	GO:0009507	Chloroplast	144/1102	183/26245	1.45e-10
CC	GO:0043227	Membrane-bounded organelle	628/1102	10408/26245	1.24e-27
CC	GO:0005886	Plasma membrane	136/1102	1557/26245	6.93e-13
MF	GO:0051082	Unfolding protein binding	36/1102	184/26245	1.83e-10
MF	GO:0048037	Cofactor binding	81/1102	792/26245	5.83e-10
MF	GO:0005506	Ion binding	91/1102	890/26245	3.17e-11
MF	GO:0016209	Antioxidant activity	72/1102	276/26245	3.88e-28
MF	GO:0009055	Electron transfer activity	86/1102	984/26245	1.59e-07

Key to legend: [A] GO-D denotes GO domain; [B] E/T denote enriched genes Vs test set: the numerator and denominator represent the number of enriched genes and test set respectively; [C] GO/Bg denote genes with GO-terms Vs back ground set: the numerator and denominator represent the number of genes for which corresponding GO-terms were assigned and the background set respectively; [D] P denote P-value (FDR). The first top GO-term in each GO-domain, eg. BP: response to stress (GO:0006950), CC: Cytoplasmic part (GO:0044444) and MF: Unfolding protein binding (GO:0051082), represent the most likely term associated to the corresponding number of enriched genes. As such, 1102, 477, and 36 genes respectively were assigned to these GO-terms.

Table S13 Databases containing potential source for candidate drought responsive genes

No.	Databases	Link or reference
1	**Genome database:**	
	Phytozome	http://www.phytozome.net/sorghum or ftp://ftp.jgi-psf.org/pub/compgen/phytozome/v9.0/Sbicolor_v1.4/; Goodstein *et al*., 2012
		http://www.gramene.org/; Ware *et al*., 2002
	Gramene	http://www.ensembl.org/index.html; Flicek et al.,
	Ensembl	2012 and 2013
2	**Sequences databases:**	
	RefSeq	http://www.ncbi.nlm.nih.gov/refseq/
	NCBI	http://www.ncbi.nlm.nih.gov/
	UniGene	http://www.ncbi.nlm.nih.gov/UniGene/; Rudd *et al*., 2003
	dbESTs	http://www.ncbi.nlm.nih.gov/dbEST/; Boguski *et al*., 1993
	Uniprot	http://www.uniprot.org/
3	**Protein domain databases**	
	Uniref	www.uniprot.org/help/uniref
	Uniprot	http://www.uniprot.org/
	Swissprot	http://www.expasy.ch/sprot/
		http://www.ebi.ac.uk/swissprot/
	Interoroscan	https://www.ebi.ac.uk/interpro/
	Signal peptides	http://www.signalpeptide.com/
4	**Expression databases:**	
	Gene Expression Omnipus (GEO): experimental genome microarray data, RNA-seq data	http://www.ncbi.nlm.nih.gov/geo/

Table S14 Relevant tools for identification of the candidate gene

Ser. #	Tools	Links or references
1	alignment tools:	
	Blast,	http://blast.ncbi.nlm.nih.gov/Blast.cgi
	Blat,	http://genome.ucsc.edu/FAQ/FAQblat.html
	est2genome	http://bioinfo.nhri.org.tw/cgi-bin/emboss/est2genome
	exonerate	http://www.csc.fi/english/research/sciences/bioscience/programs/exonerate/index_html
	ClustalW	http://www.genome.jp/tools/clustalw/
	ClustalX	http://www.clustal.org/clustal2/
2	Gene structure prediction tools:	
	Augustus,	http://augustus.gobics.de/binaries/
	est2genome	ftp://ftp.hgc.jp/pub/mirror/ebi/software/exonerate/
	exonerate, and	http://bioinfo.nhri.org.tw/cgi-bin/emboss/est2genome
	PASA	http://www.evidencemodul
	Evidence Moduler (EVM),	http://evidencemodeler.sourceforge.net/
3	Gene enrichment analysis tools:	
	AgriGO	http://www.agrigo.org/
	Blast2go	http://www.blast2go.de
	MeV,	http://www.tm4.org/mev.html
	Genevestigator,	https://www. genevestigator. ethz. Ch/
4	Genome viewer tools:	
	Galaxy,	http://usegalaxy.org; https://main.g2.bx.psu.edu/
	Gbrowse	http://gmod.org/wiki/GBrowse/tool_data;
	USCS-Gbrowse	http://genome.ucsc.edu/
	Biomart-ensemble,	http://www.ensembl.org/info/data/biomart.html;
	Biomart-Gramene	http://archive.gramene.org/biomart/martview/334378ea281f8c0071af7d45f4ab01f9
	Biomart Phytozome	http://www.phytozome.net/biomart/martview/6ae06d47637222933cd94d5beb507c71
5	Expression profiling analysis tools:	
	Multivariate Experiment Viewer (MeV)	http://www.tm4.org/mev.html
6	Sequence quality filtering tools:	

RepeatMasker	http://www.repeatmasker.org/
Statistical analysis tools:	
R programming,	http://www.r-project.org/
MeV	http://www.tm4.org/mev.html
7 Programming tools:	
linux OS	http://www.gnu.org/
Perl,	http://www.perlmonks.org/
Python,	http://www.tutorialspoint.com/python/python_tools_u
R, programming modules,	tilities.htm
Bioperl	http://www.r-project.org/
Biopython	www.bioperl.org/
	www.biopython.org/;http://biopython.org/wiki/Main_
	Page

Table S15 Functional description of sorghum drought related metabolic pathways

Pathway	Enzyme	Enzyme ID	Pathway map ID	Pathway ID	gene (id) involved
Aminoacyl-tRNA biosynthesis	ligase	EC:6.1.1.16	map00970	KO:K01883	*genes
Cysteine and methionine metabolism	Dioxygenase (iron(II)-requiring)	EC:1.13.11.54	map00270	KO:K08967	Sb01g046360
Drug metabolism - other enzymes	ali-esterase	EC:3.1.1.1	map00983	*KO	novel
Glucosinolate biosynthesis	CYP79A1, tyrosine N-mono-oxygenase	EC:1.14.13.41	Map00966 (sbi00966)	KO:K13027	Sb01g001200
Glycerophospholipid metabolism	kinase (ATP dependent)	EC:2.7.1.107	map00564	KO:K00901	Sb01g032250
Glycerolipid metabolism	kinase (ATP dependent)	EC:2.7.1.107	map00561	KO:K00901	Sb01g032250
Phosphatidylinositol signalling system	kinase (ATP dependent)	EC:2.7.1.107	map04070	KO:K00901	Sb01g032250
Nicotinate and nicotinamide metabolism	kinase	EC:2.7.1.23	map00760	KO:K00858	Sb09g019130
Oxidative phosphorylation	Cytochrome c oxidase subunit 1 (Oxidase); Inorganic diphosphatase	EC:1.9.3.1; EC:3.6.1.1	Map00190 map00190	KO:K02256 KO:K02256	Sb09g022400, COX1; **genes
Pyrimidine metabolism	RNA polymerase	EC:2.7.7.6	map00240	**KO	***genes
Purine metabolism	RNA polymerase; adenylpyrophosphatase	EC:2.7.7.6; EC:3.6.1.3	Map00230 map00230	**KO KO:K01509	*** novel
Pantothenate biosynthesis	BCAT	EC:2.6.1.42	sbi00770	KO:K00826	****genes
VLIB	BCAT	EC:2.6.1.42	sbi00290	KO:K00826	****genes
VLID	BCAT	EC:2.6.1.42	sbi00280	KO:K00826	****genes

Key to legend:
*KO K01044 carboxylesterase 1; K03927 carboxylesterase 2; K03928 carboxylesterase; K15743 carboxylesterase 3/5
**KO KO:K03006;KO:K02999; KO:K03002; KO:K03006; KO:K03018; KO:K03021; KO:K03043; KO:K03046
*genes Sb01g047380; Sb02g032450
**genes Sb09g001530; Sb10g009880; Sb09g004450; Sb09g021610; Sb01g022340; Sb04g036230; Sb04g005710; Sb04g034340; Sb03g013530; Sb03g040910
***genes Sb05g019520; Sb03g017630; Sb03g020184; Sb04g001790; Sb04g009491; Sb05g019520; Sb06g021120; Sb07g003680; Sb09g027223; Sb09g027230; Sb10g006995
****genes Sb04g010240; Sb06g025140; and Sb09g008180

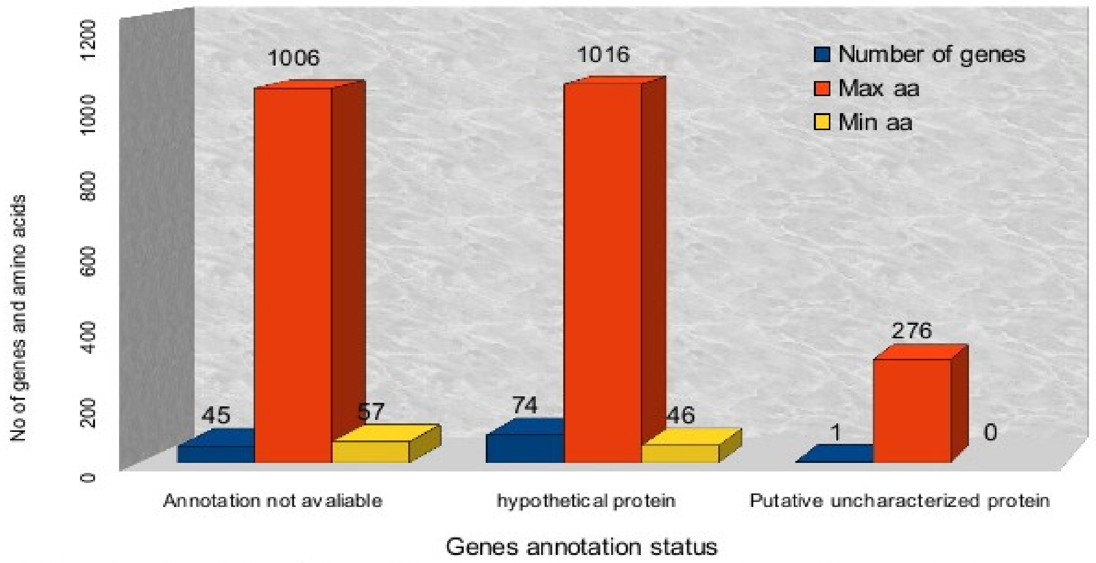

Figure S1. Identified genes by mapping to existing annotation and their characterization status

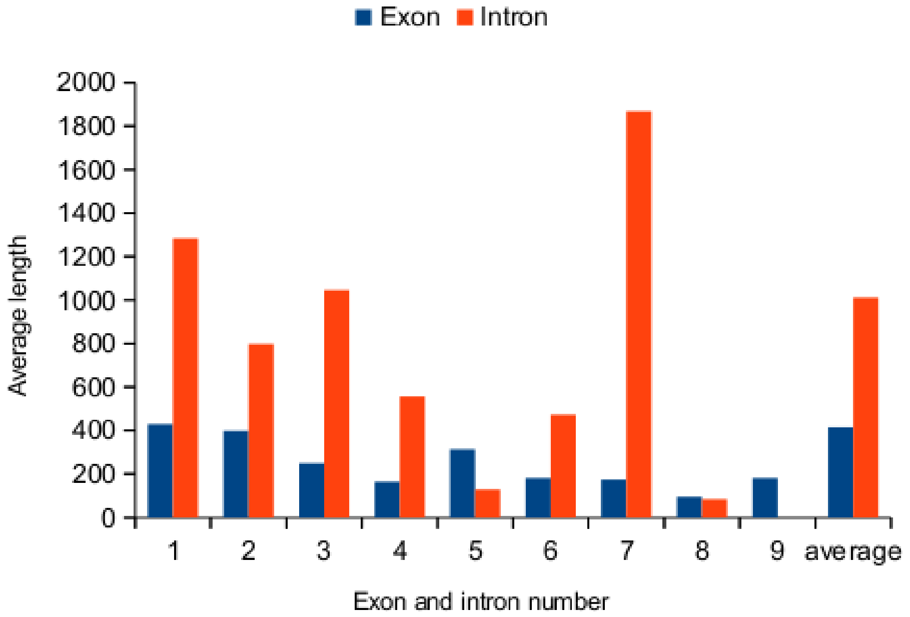

Figure S2 Pattern of exon and intron number and the average length for novel identified genes.

Key to legend for exon and intron number: 1=exon1, intron1; 2=exon2, intron2; 3=exon3, intron3; 4=exon4, intron4; 5=exon5, intron5; 6=exon6, intron6; 7=exon7, intron7; 8=exon8, intron8; 9=exon9, intron9 and average = exon total average; intron total average.

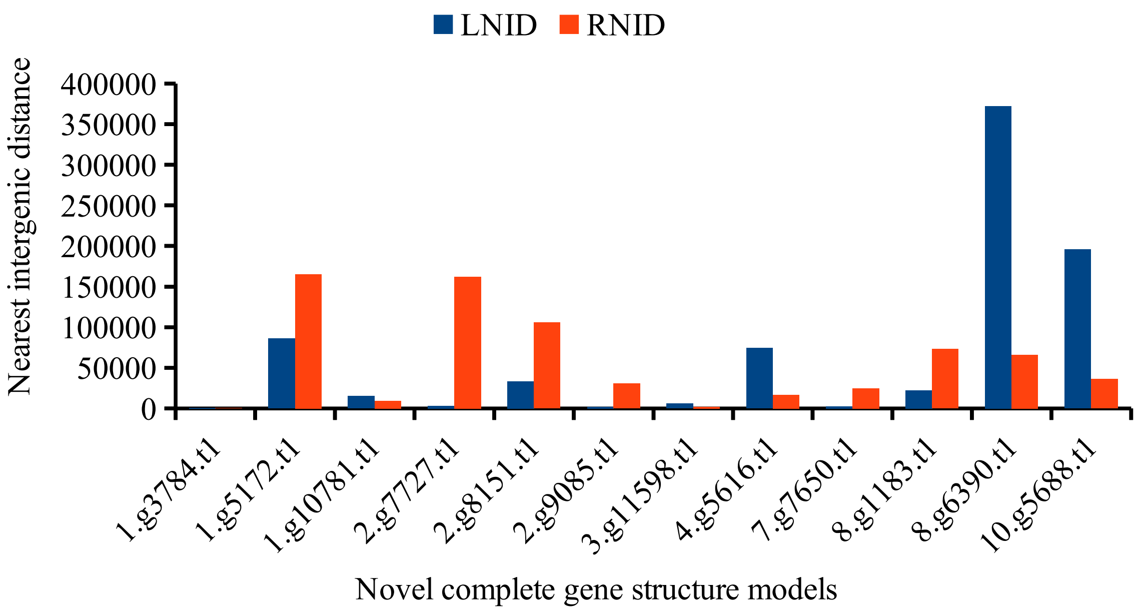

Figure S3 Nearest intergenic distances.

Key to legend: RNID (right nearest intergenic distance) and LNID (left nearest intergenic distance). The intergenic distances between the NGSM and the nearest existing neighbour gene were described based on the fourteen NGSMs known to have complete gene structure. The average RNID represents 57687 bases long while the average LNID represents 67754 bases long.

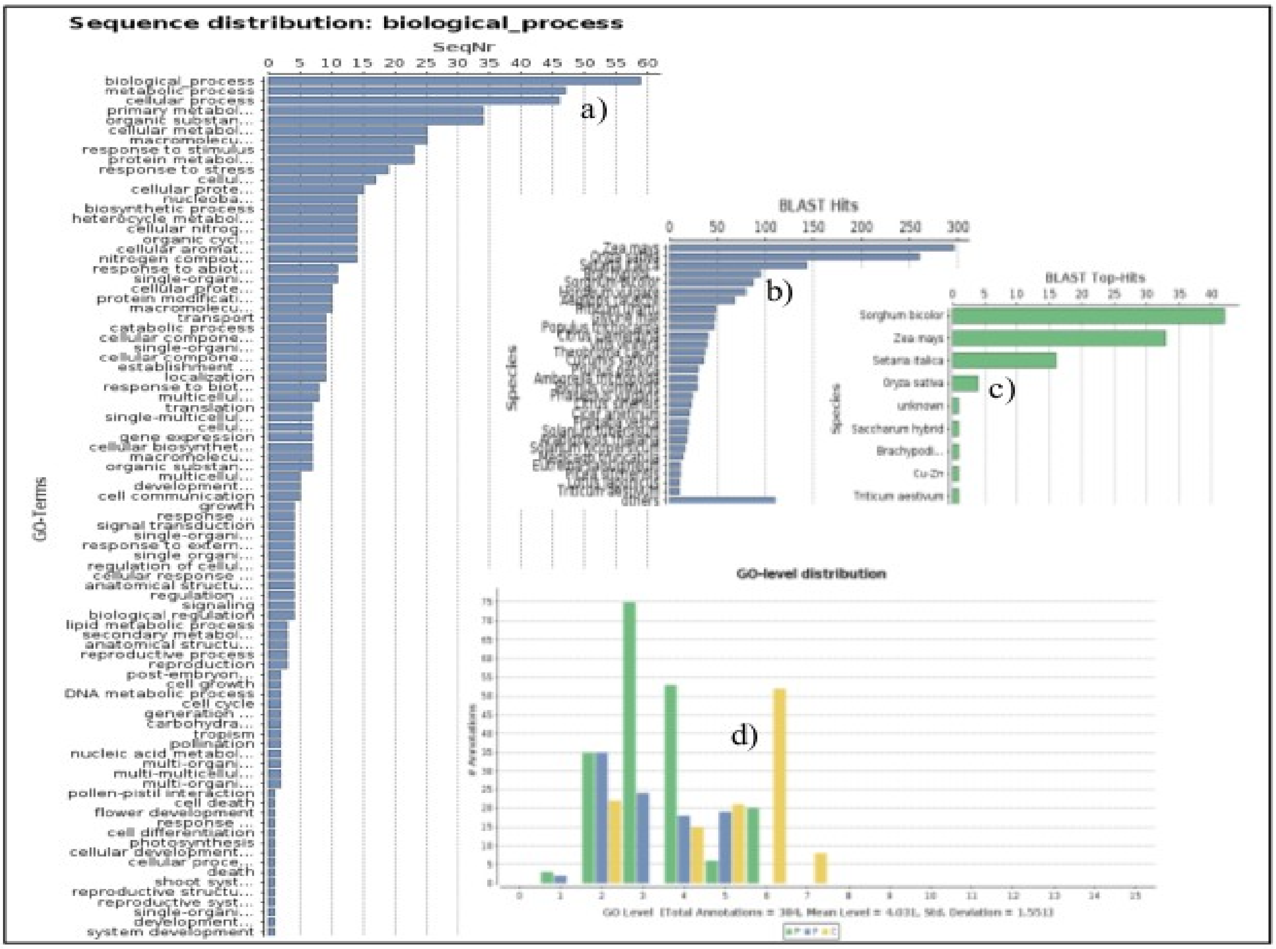

Figure S4 GO annotation based on blasting and mapping to non-redundant databases. GO annotation based on blasting and mapping sequences to non-redundant databases based on balst2go: a) patterns of sequences distribution based on blast hits associated to the GO-terms for the biological process; b) Species specific score for the total blast-hit; c) species specific blast top hits and d) GO-level distribution of annotations

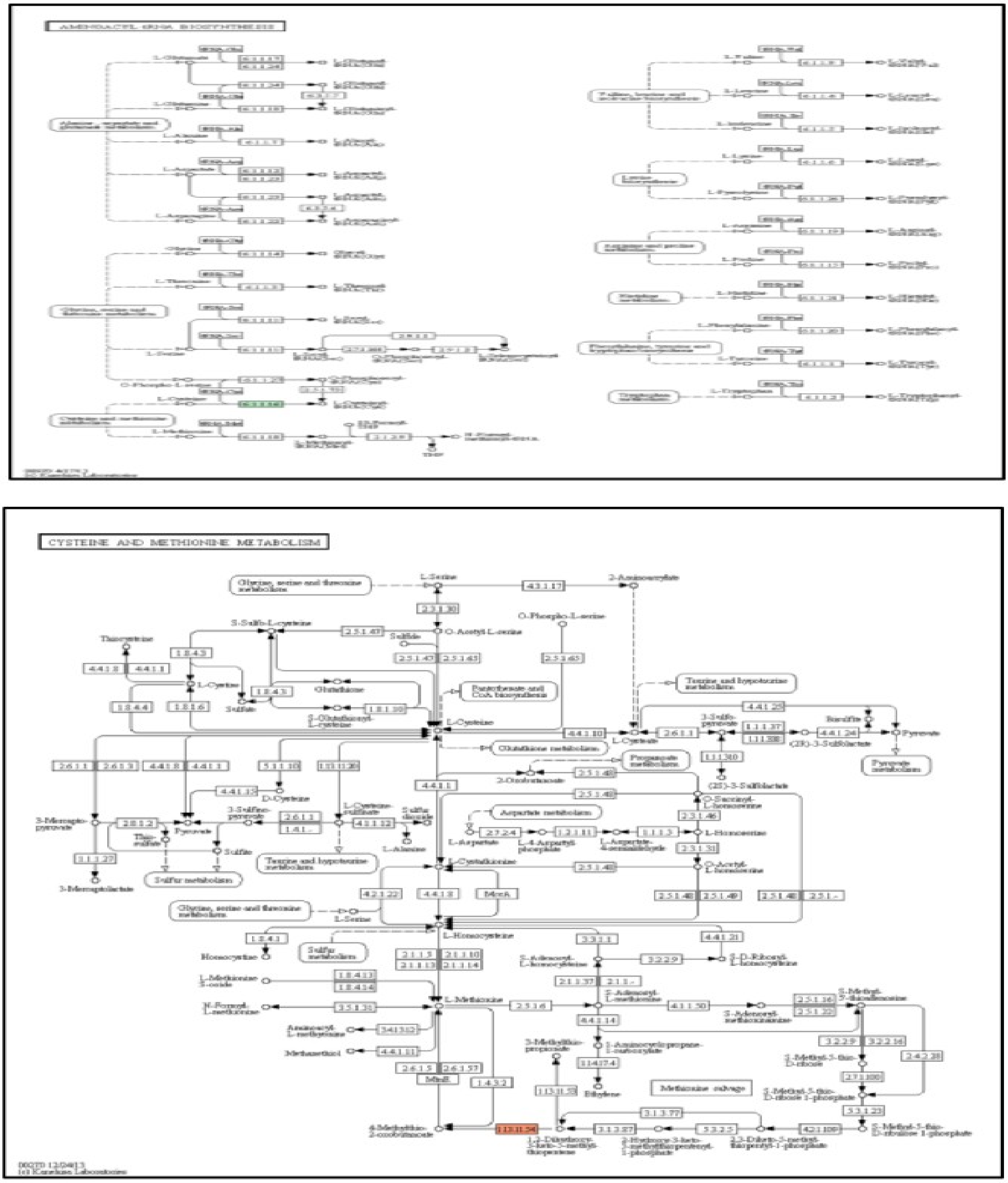

Figure S5 Aminoacyl-tRNA biosynthesis (upper) and Cysteine and methionine metabolism (lower).

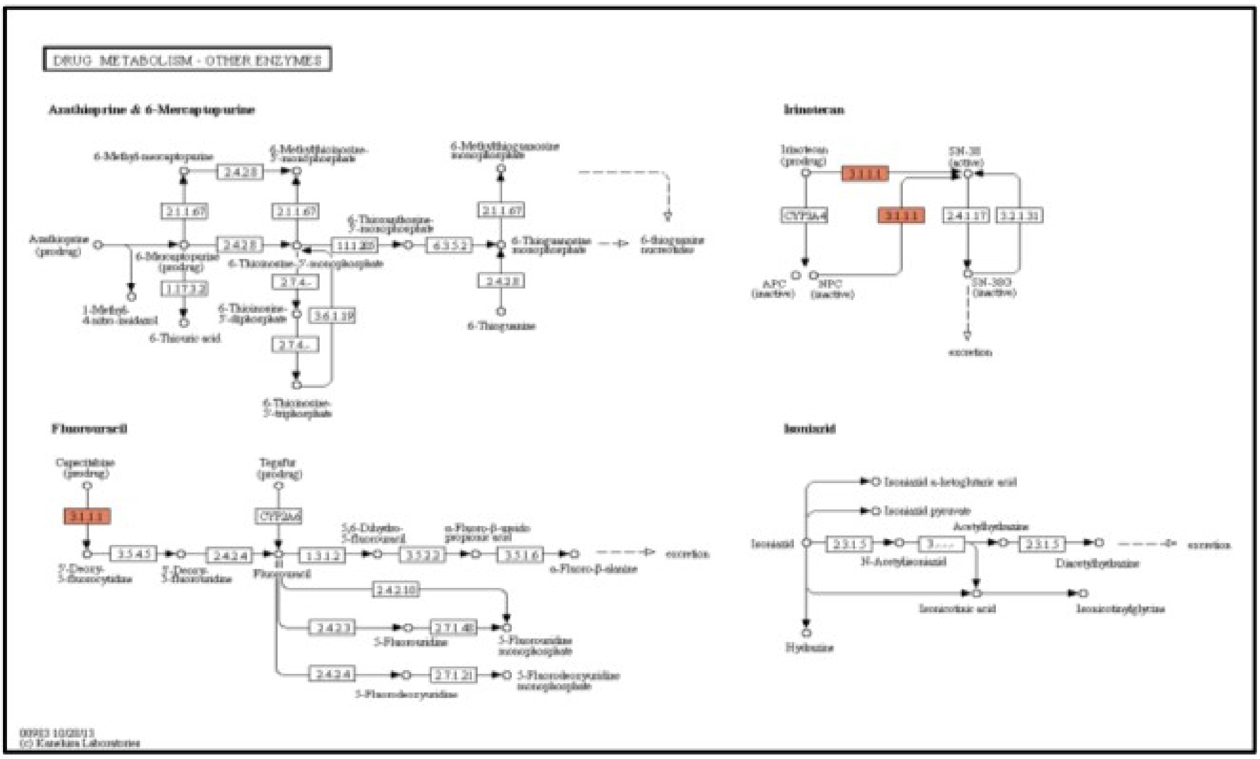

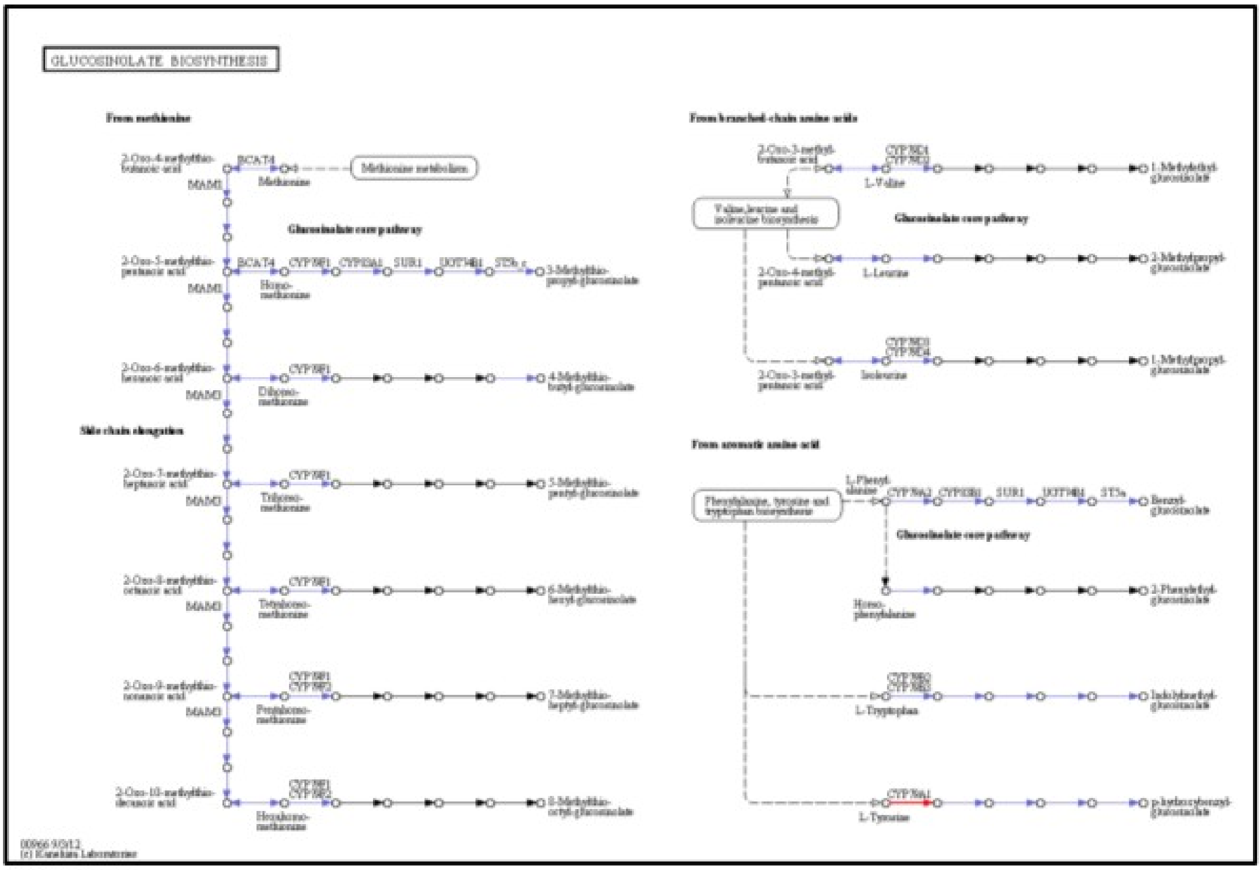

Figure S6 Drug metabolism - other enzymes (upper) and Glucosinolate biosynthesis (lower).

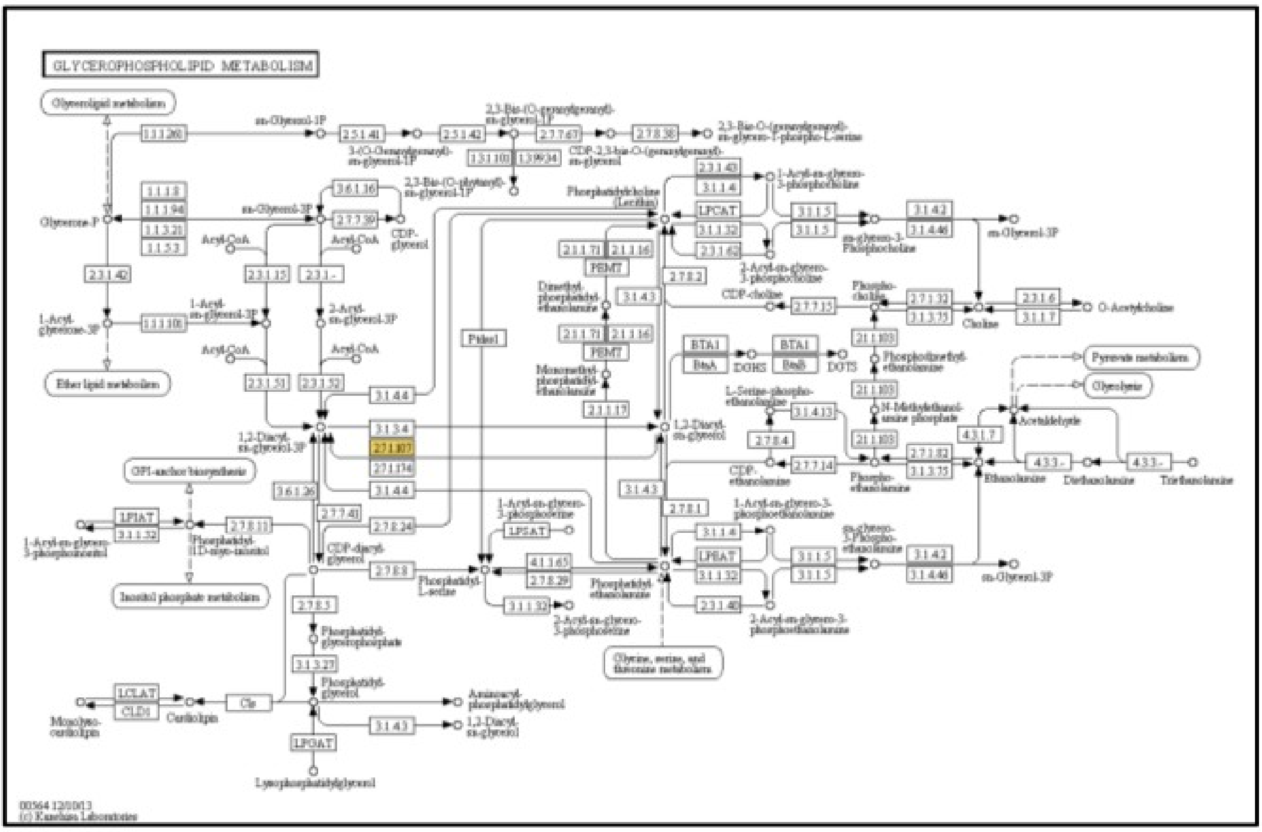

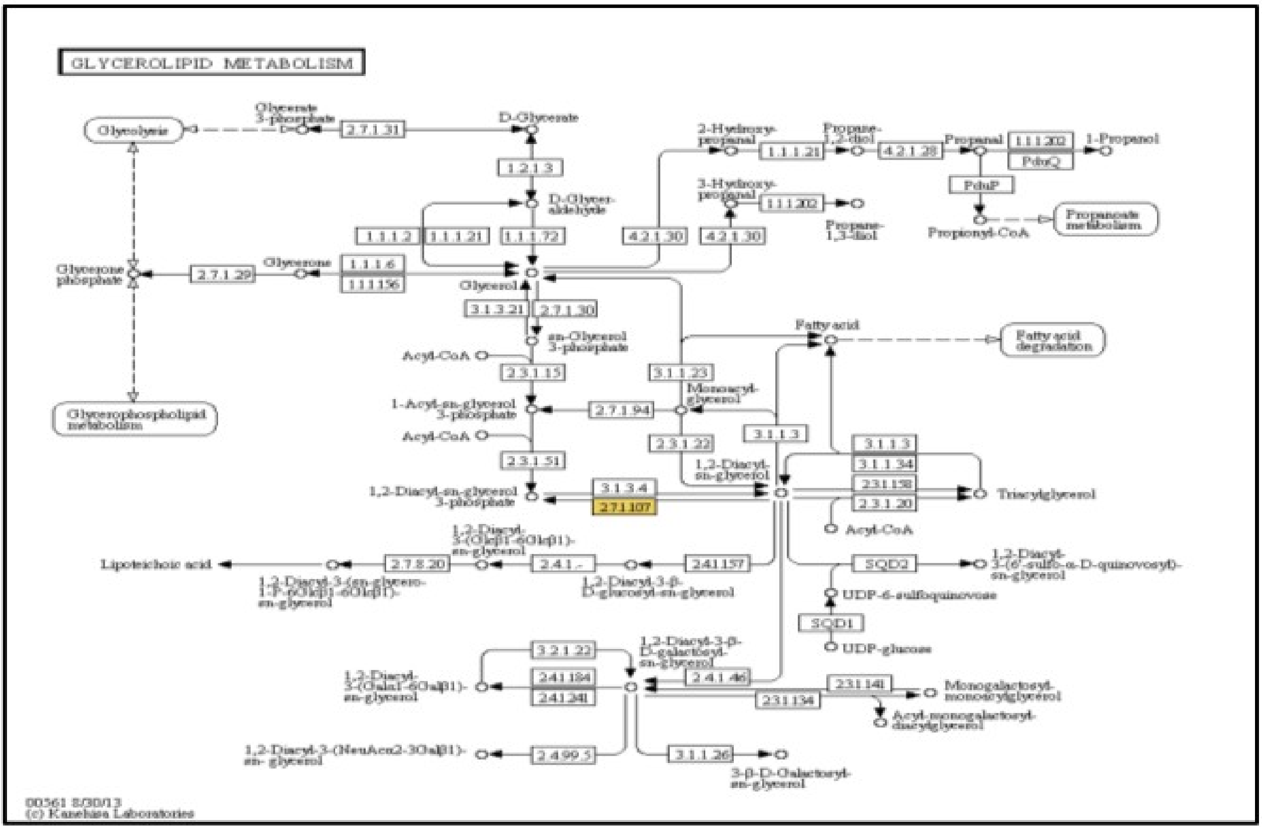

Figure S7 Glycerophospholipid metabolism (upper) and Glycerolipid metabolism (lower).

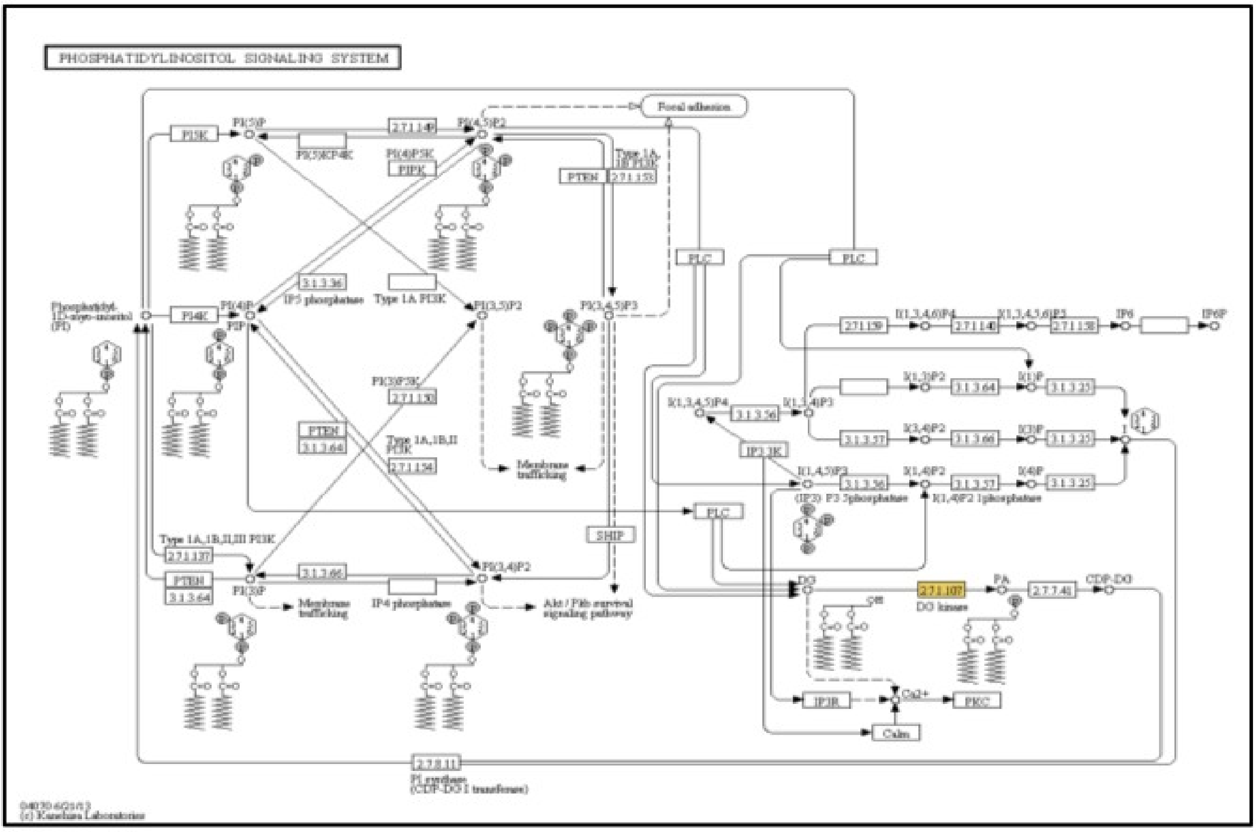

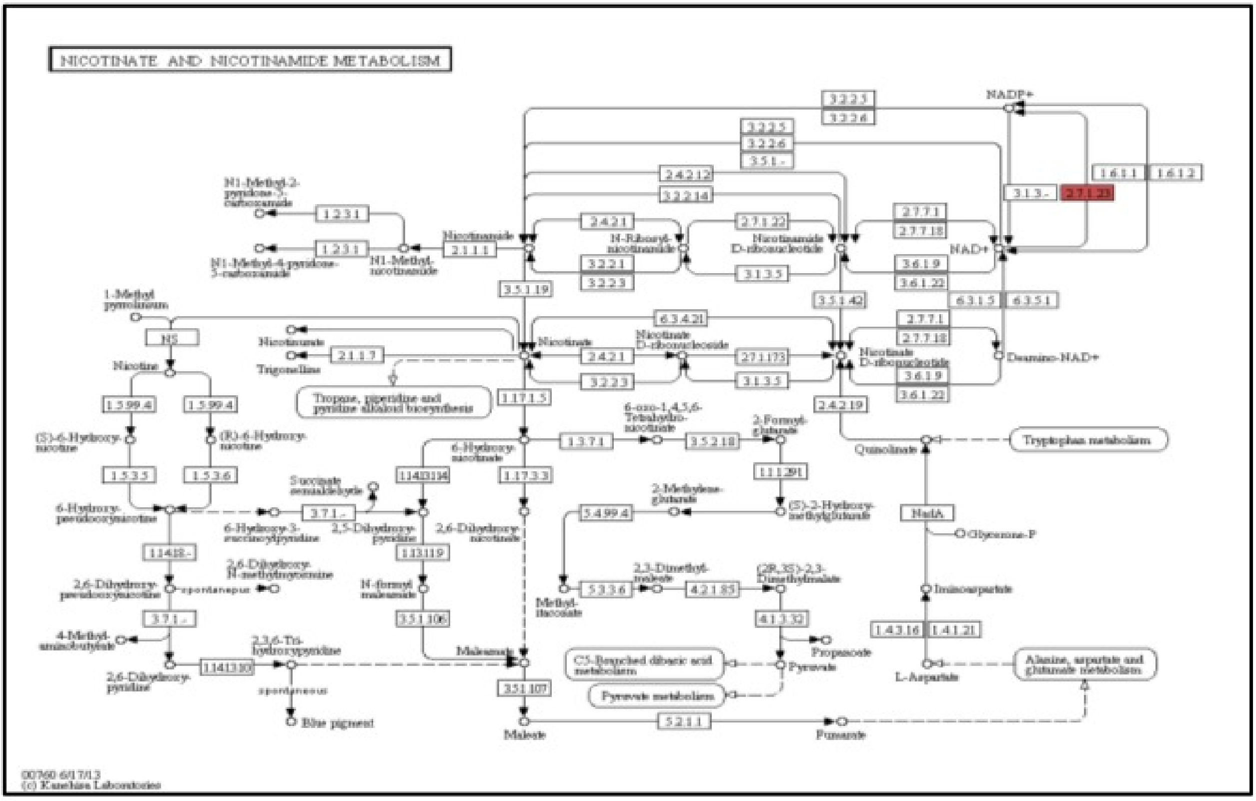

Figure S8 Phosphatidylinositol signalling system (upper) and nicotinate and nicotinate metabolism (lower).

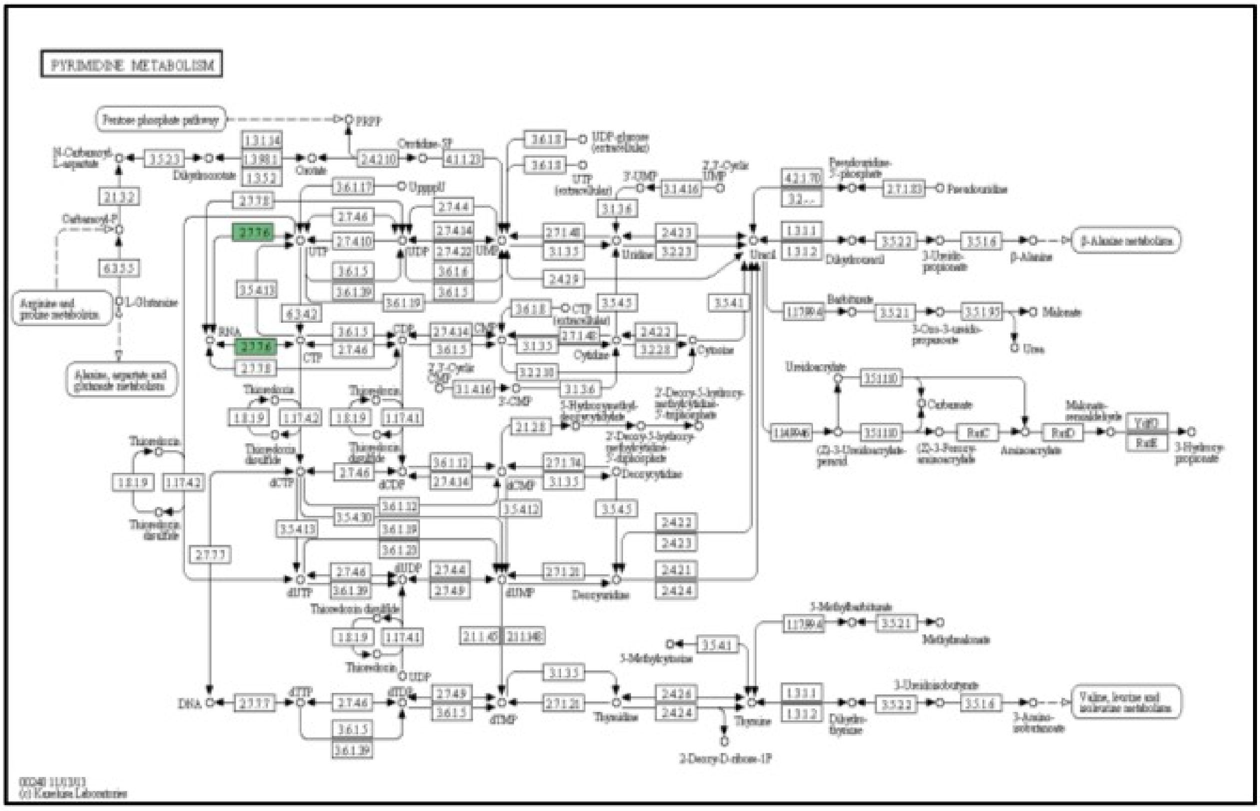

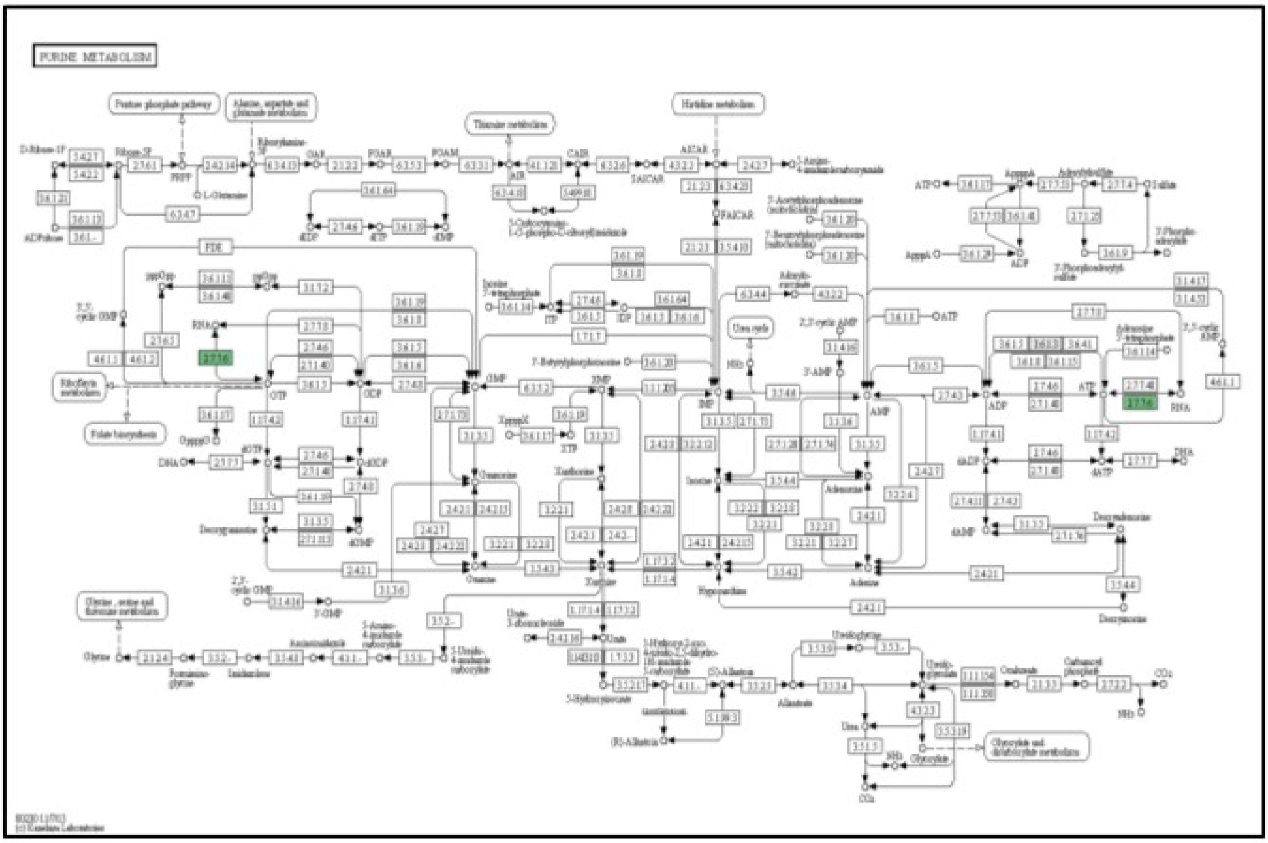

Figure S9 Pyrimidine metabolism (upper) and purine metabolism (lower).

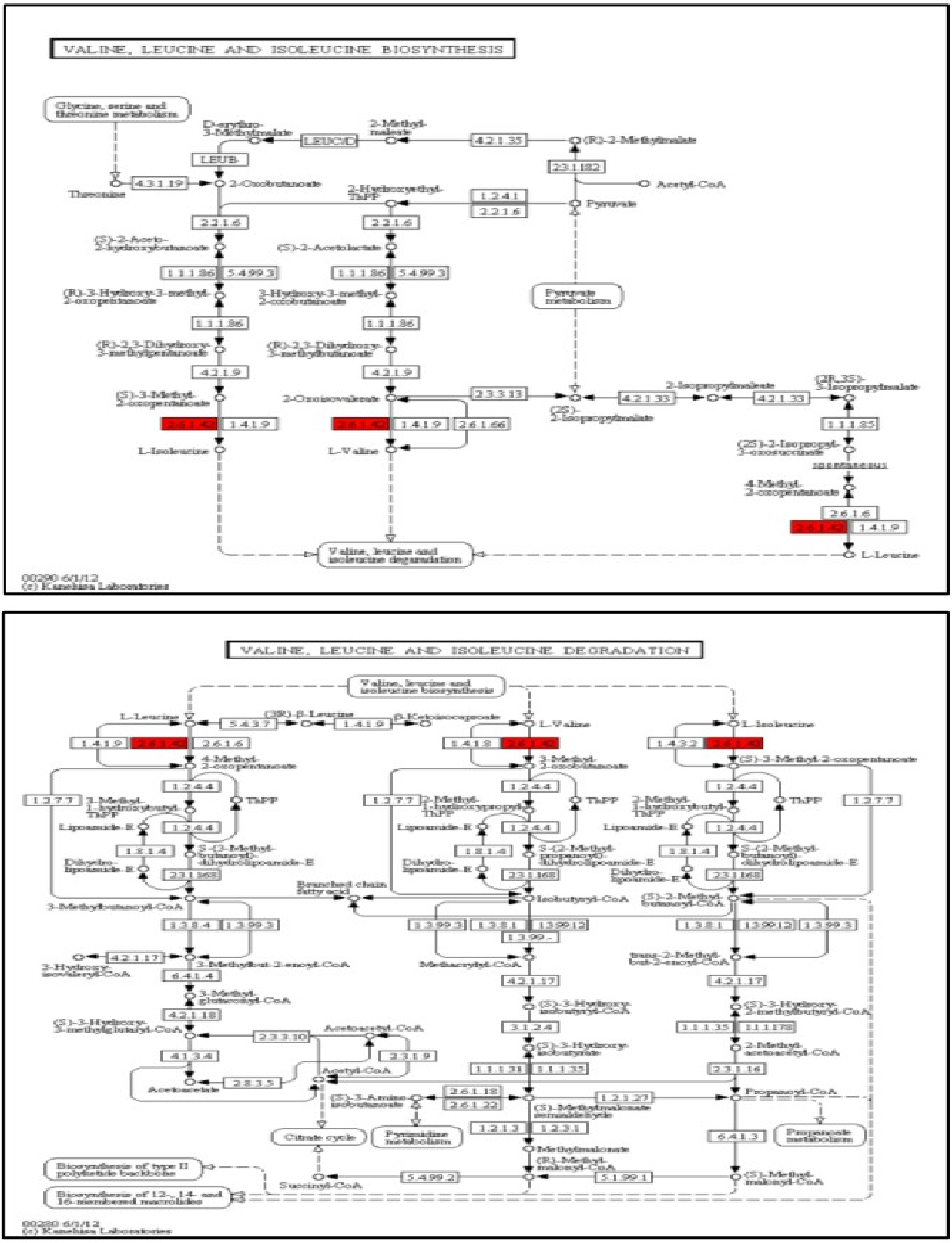

Figure S10 Valine, Luecine and Isoleucine Biosynthesis (upper) and Valine, Luecine and Isoleucine degradation (lower).

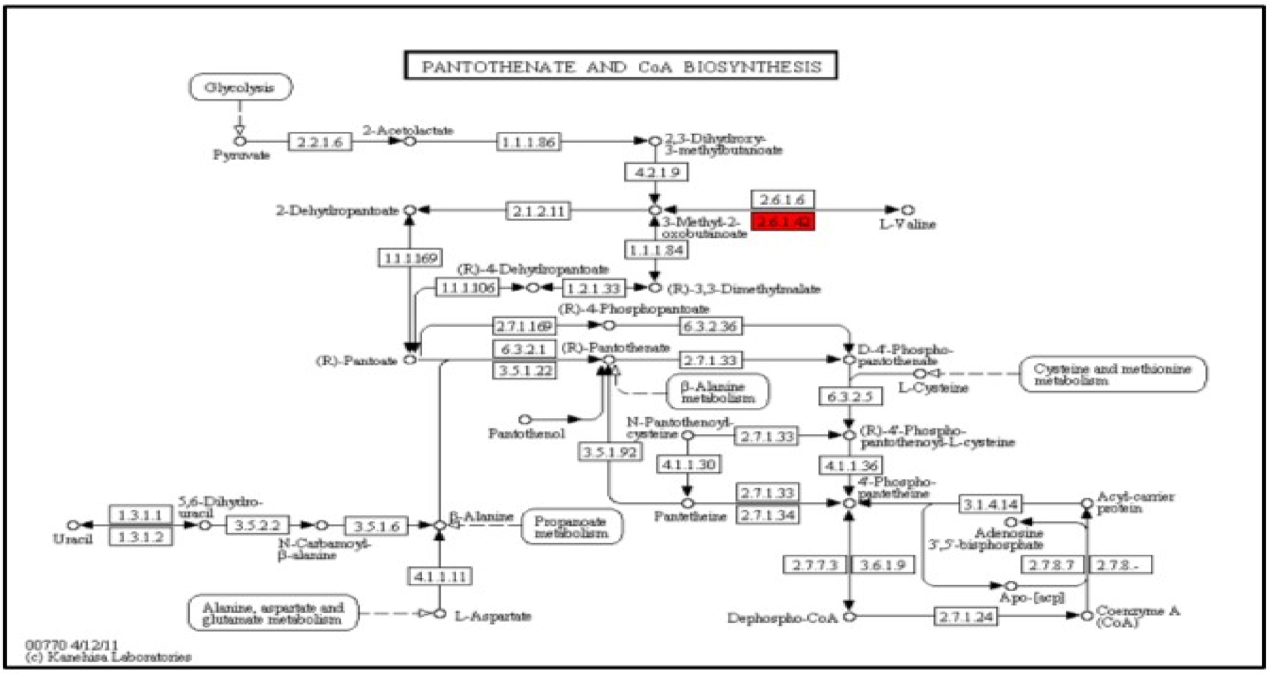

Figure S11 Pantothenate and CoA biosynthesis.

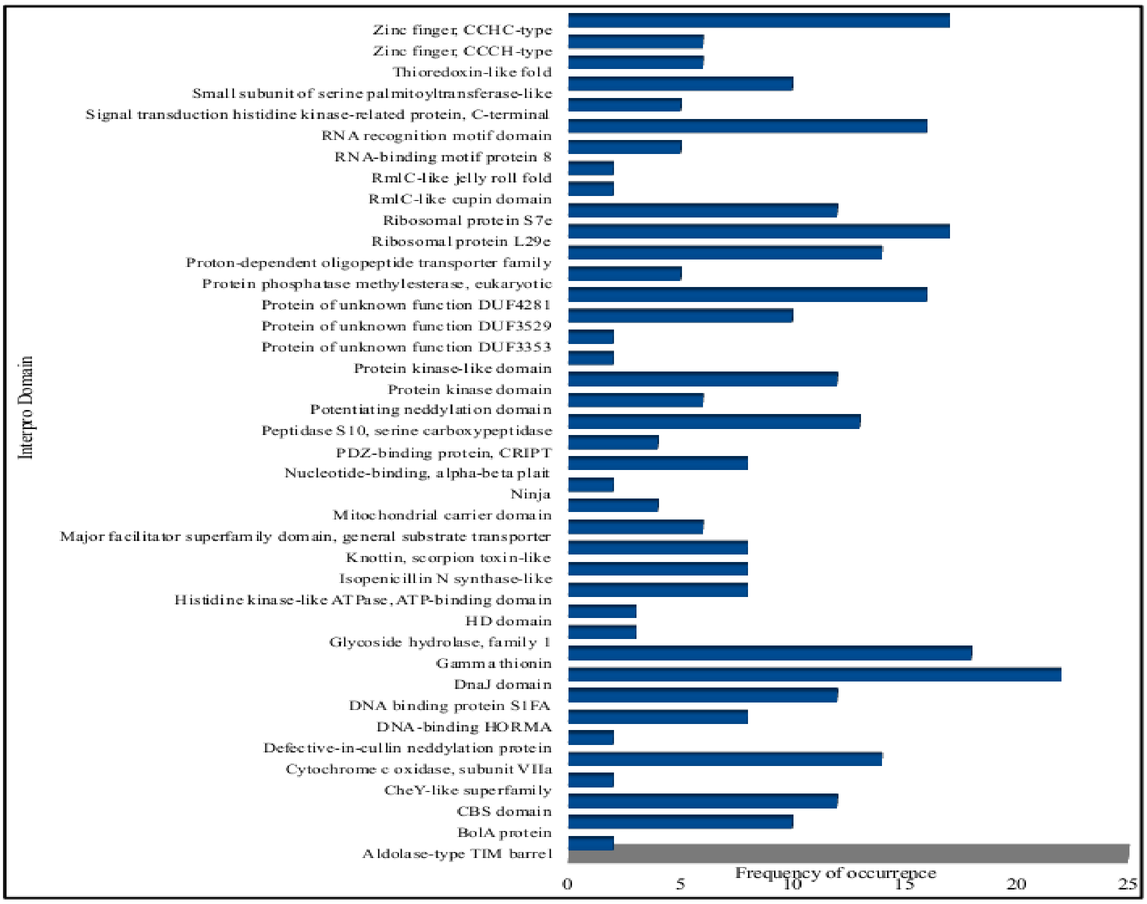

Figure S12 Description of interpro-domain analysis: List of protein signatures identified.

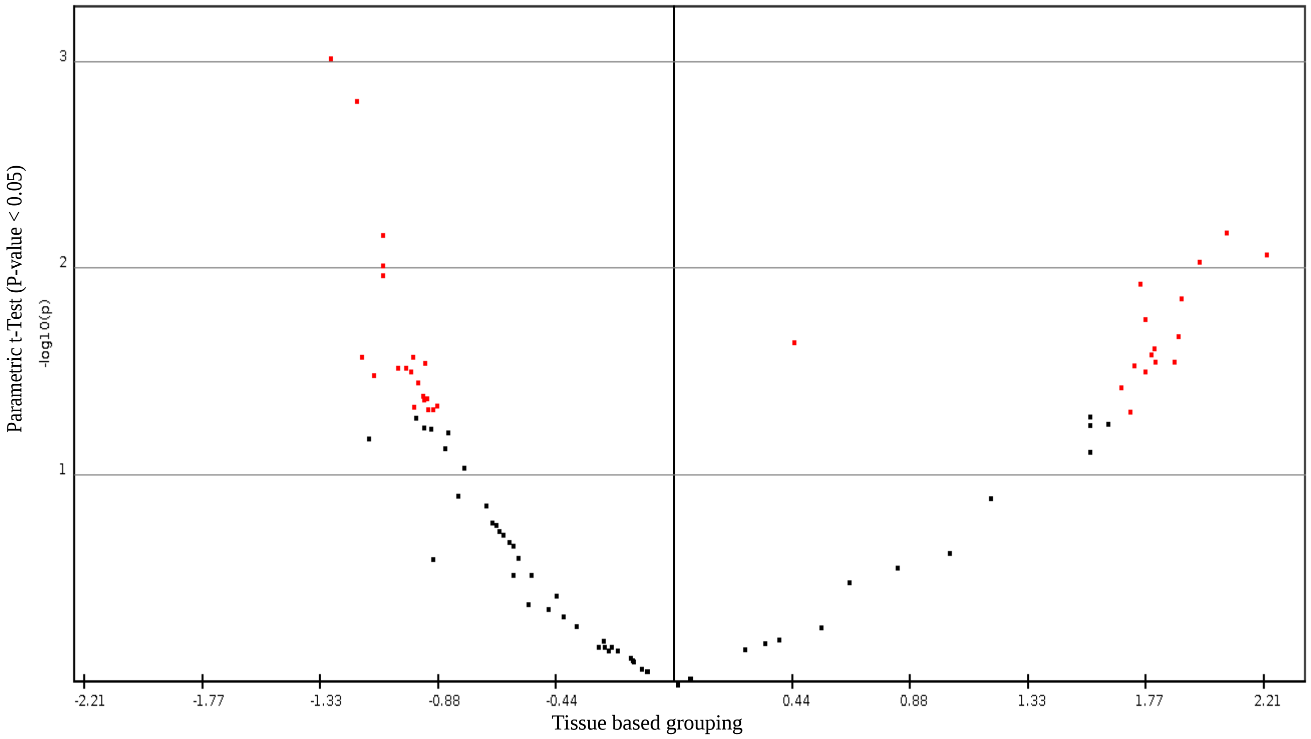

Parametric t-Test (P-value < 0.05)
-log10(p)
3
2
1
-2.21
-1.77
-1.33
-0.88
-0.44
0.44
0.88
1.33
1.77
2.21
Tissue based grouping

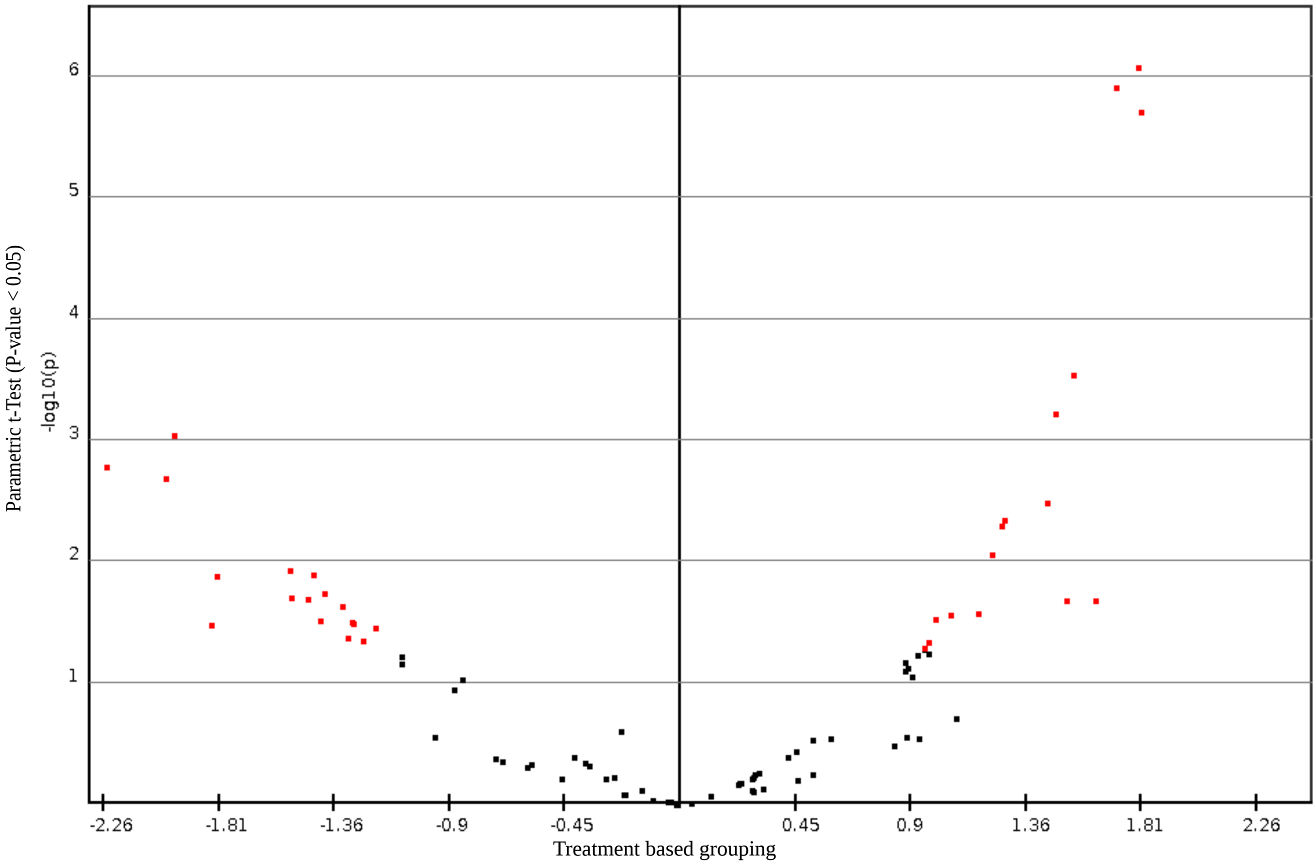

Figure S13. **Volcano plots for expression profiles based on sorghum drought responsive genes.** This figure shows volcano plots with differential expression of genes with most significant at the top of the plot. Volcano plot (a) represents unpaired t-test based on the evaluation of tissue type contributing to the gene expression and (b) on the evaluation of treatment effect on the experimental samples. The red dots indicate a statistical significance for the up and down-regulated genes at the fold-changes (two) above which all genes have $p < 0.01$ and below which $p > 0.01$.

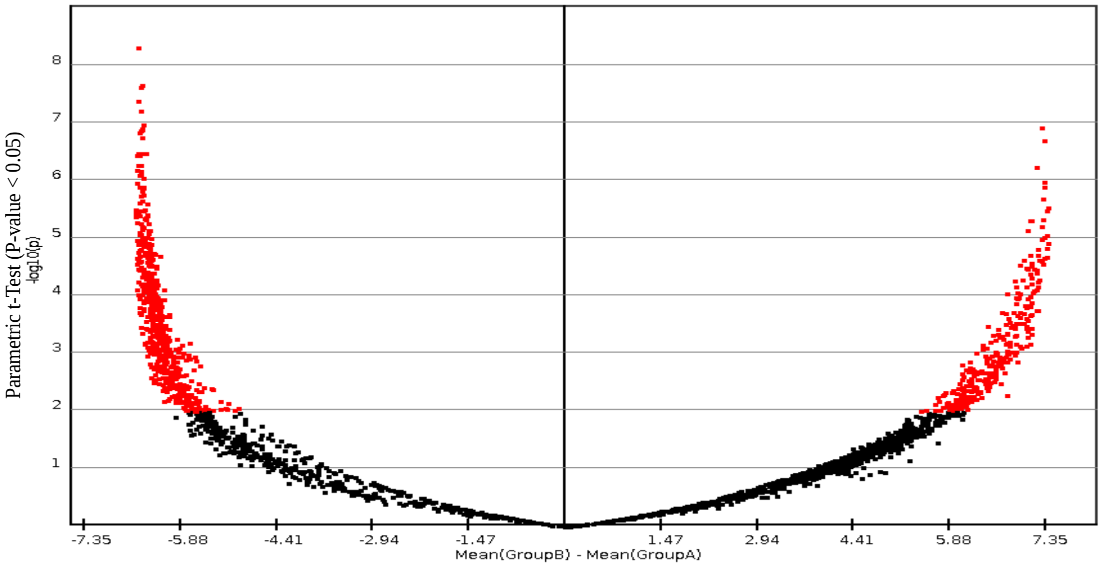

Tissue based grouping

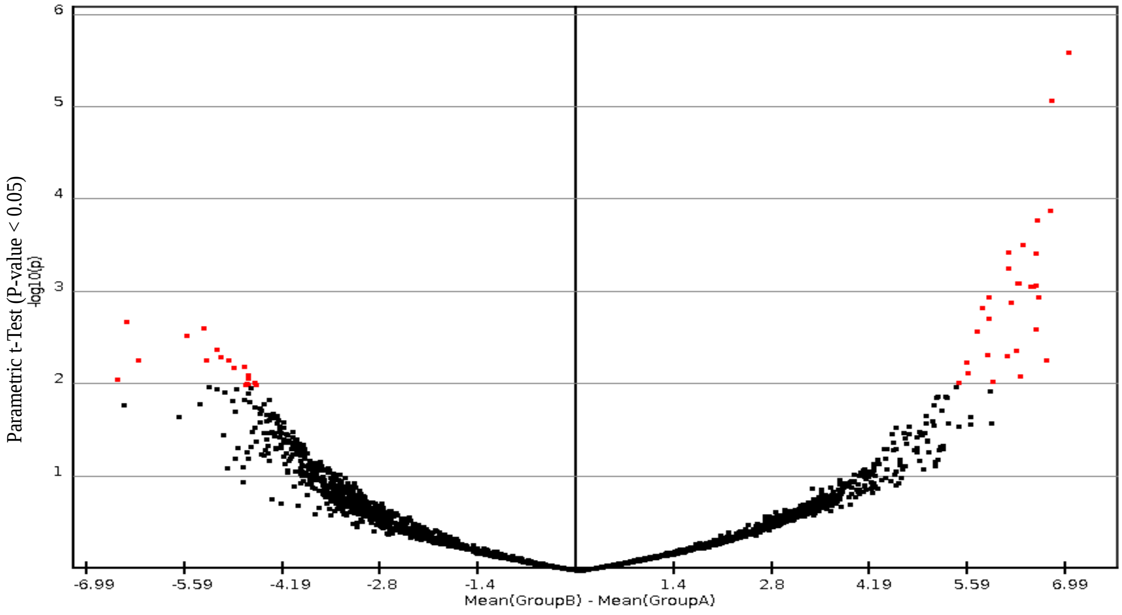

Treatment based grouping

Figure S14 Volcano plots showing expression profiles based on based on maize expression data.

This figure describes volcano plot showing differential expression of genes with the most significant genes at the top of the plot. The red dots indicate genes-of-interest that display both large-magnitude of fold-changes (the change in mean values of group A and group B, in our case, x-axis) and a fairly high value of statistical significance (-log10 of p-value, y-axis). The upper line

across the plot shows a point where $p = 0.01$ (i.e. where the fold-change is equal to two (log2 = 1) above which lie all genes having $p < 0.01$ and below which having $p > 0.01$. Volcano plots a) represent unpaired t-test based on treatment grouping, b) depicts the gene expression pattern based on between subject variance with tissue based grouping of samples. The plot shows higher number of genes expressed under drought condition in tissue based grouping with more down-regulated tissue specific genes than in treatment based grouping which shows not only relatively fewer number of genes expressed in total under the same stress condition but also relatively less down-regulated genes.

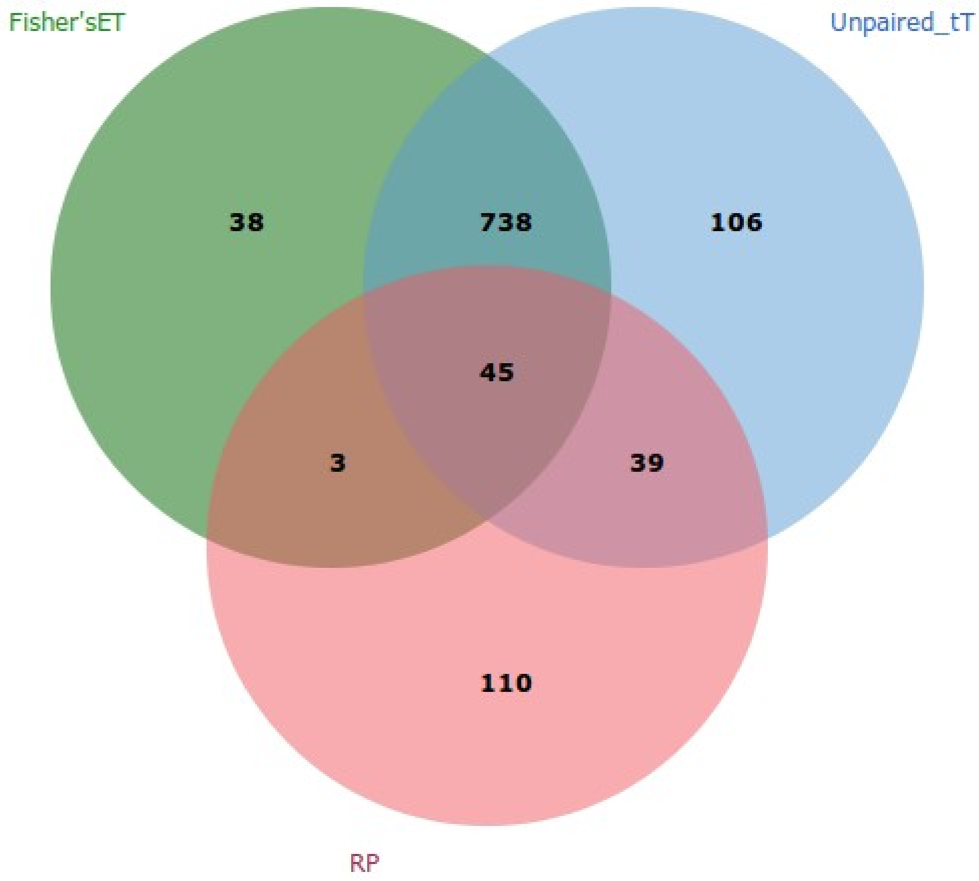

Figure S15 Venn diagram showing distribution of significantly expressed genes.

Key to legend: Fisher's ET = Fishers Exact Test; Unparired tT = Unpaired t test and RP = Rank product. The figure illustrates the number of statistically significantly expressed genes identified by each statistical model which is equivalent to the sum of values in each respective circle. An overlapping shows the number of significantly expressed genes supported by multiple models. The number at the centre represents a total number of expressed genes supported by all statistical models.

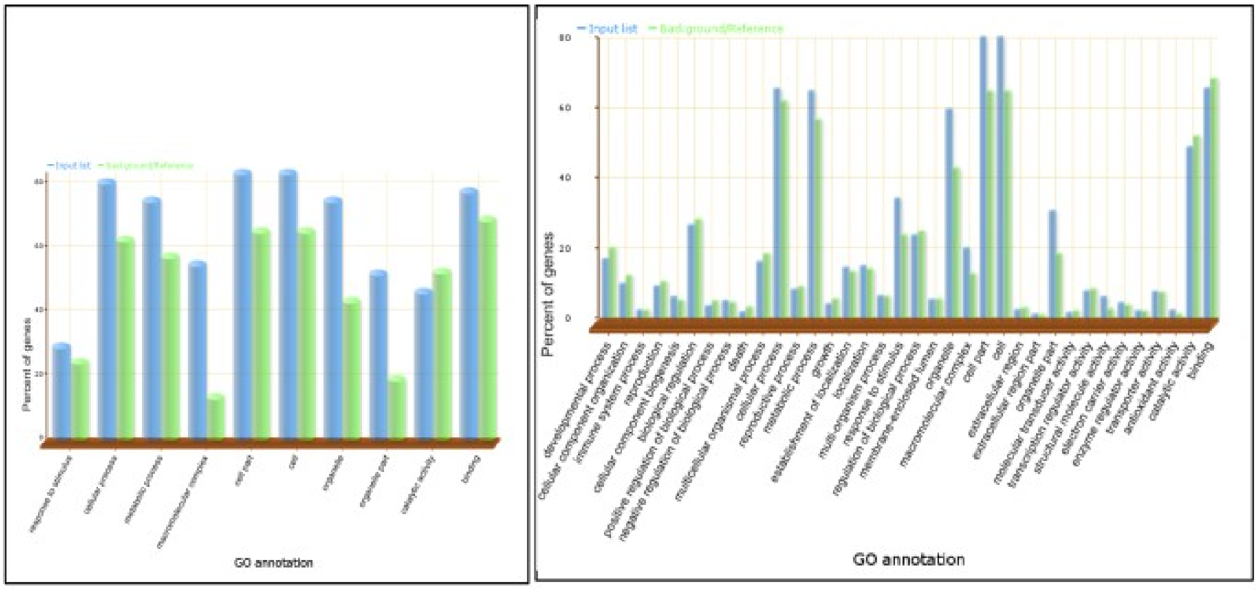

Figure S16 Sorghum % GO-terms assigned genes identified from maize orthologs. Significantly expressed 45 genes supported by all statistical models based on analysis of gene-expression are shown (left) and significantly expressed total 1079 genes supported by individual statistical models based on analysis of gene-expression are also shown (right). The percentage of sorghum genes identified from maize orthologs that were assigned to different GO-terms associated to drought responses are also shown on gene expression in maize reproductive and Leaf meristem tissue.

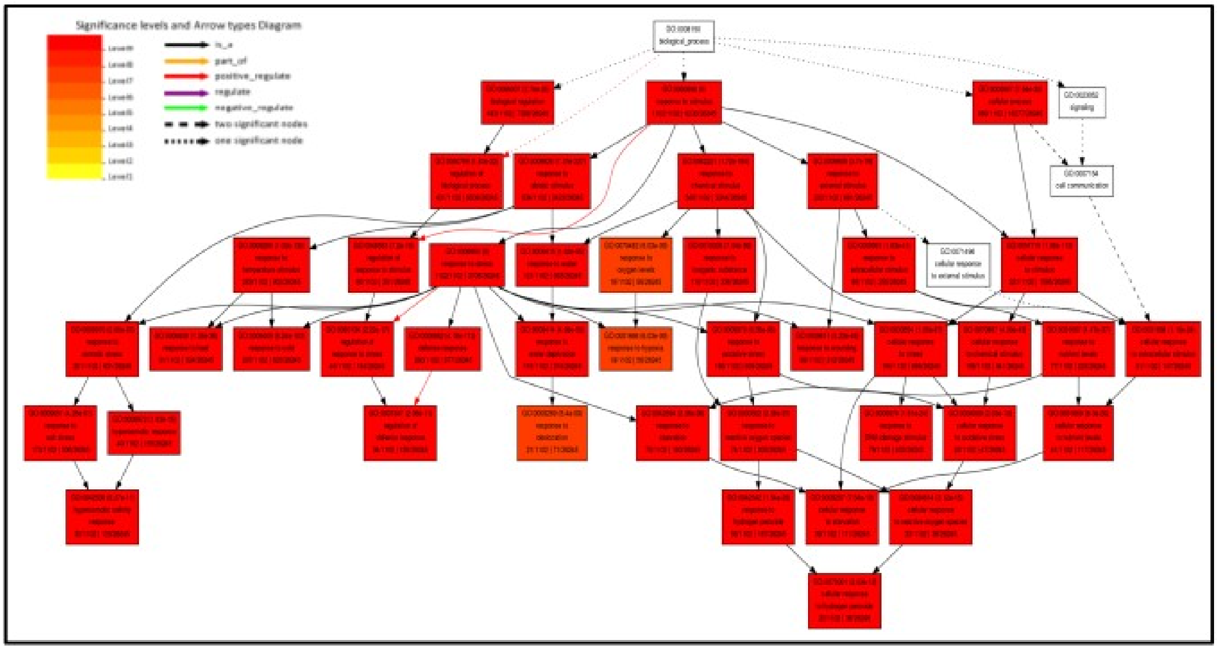

Figure S17 Mapping of GO terms related to responses to drought stress based on the biological process of the genes identified using orthology groups. This figure represents significantly overrepresented GO-terms most related to drought tolerance selected based on tree traversing mode using AGRIGO software package. The GO term descriptors that related to drought tolerance included but not limited to "response to drought stress", "response to desiccation tolerance", "response to water deprivation", "cellular response to drought stress", "cellular response to desiccation" and "cellular response to water deprivation". Drought stress regulated genes were represented as an expression of mRNAs with more than two-fold changes under differential conditions. The number inside the coloured box represent GO accessions, p-values, number of enriched genes in the test set per total number of genes involved in the test set and those in the sorghum database associated with GO-term per total number of genes in the database involved in the background set of the GO-term.
